# Two new glucosides from the pellicle of the walnut (*Juglans regia*)

**DOI:** 10.1007/s13659-012-0009-0

**Published:** 2012-06-22

**Authors:** Le Cai, Chuan-Shui Liu, Xiao-Wei Fu, Xiao-Jing Shen, Tian-Peng Yin, Ya-Bin Yang, Zhong-Tao Ding

**Affiliations:** Key Laboratory of Medicinal Chemistry for Nature Resource, Ministry of Education, School of Chemical Science and Technology, Yunnan University, Kunming, 650091 Yunnnan, China

**Keywords:** walnut, *Juglans regia*, pellicle, *α*-tetralonyl glucoside, dihydrophaseic acid glucoside

## Abstract

A new *α*-tetralonyl glucoside, 6′-*O*-acetyl-juglanoside E (**1**), and a new dihydrophaseic acid glucoside, dihydrophaseic acid 1-*O*-(6-*O*-acetyl)-glucopyranoside (**2**), together with two known ones, juglanoside E (**3**) and dihydrophaseic acid (**4**), were isolated from the pellicle of the walnut (*Juglans regia*). The structures of the new compounds were elucidated by comprehensive spectroscopic analysis, including IR, HRESIMS, 1D and 2D NMR data.

**Electronic Supplementary Material:**

Supplementary material is available for this article at 10.1007/s13659-012-0009-0 and is accessible for authorized users.

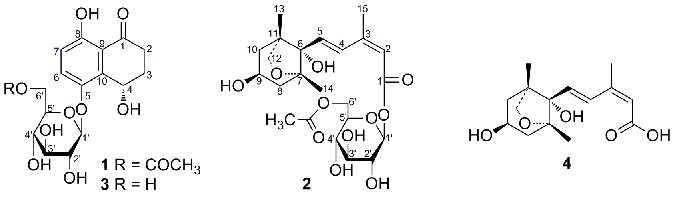

## Introduction

The *Juglans* genus (family Juglandaceae) comprises several species and is widely distributed all over the world. The walnut tree (*Juglans regia* L.), a well-known member of this genus, is cultivated commercially throughout southern Europe, northern Africa, eastern Asia, United States and western South America. In China, *Juglans regia* is not only an agricultural commodity, but can be also used for different medicinal functions. Walnuts, the seeds of *Juglans regia*, are a highly nutritious food, which have been found to be a rich source of essential unsaturated fatty acids, tocopherols,[1] hormone and strong antioxidant melatonin.[2] Mahoney et al. reported that the activity of inhibiting aflatoxigenesis in *Aspergillus flavus* located entirely in the pellicle (seed coat) of walnuts.[3] However, to our knowledge, few phytochemical investigations on the pellicle of walnuts have been undertaken.[4,5] This paper describes the separation and structural elucidation of a new *α*-tetralonyl glucoside (**1**) and a new dihydrophaseic acid glucoside (**2**), along with two known ones, juglanoside E (**3**) and dihydrophaseic acid (**4**) from the pellicle of this plant (Figure 1).

**Figure 1.** Structures of compounds **1**–**4**

## Results and Discussion

Compound **1** was obtained as white amorphous powder with mp 84.5–86.2 °C. The positive HRESIMS spectrum showed the $[M + Na]^+$ ion peak at *m/z* 421.1112 (calcd 421.1110) in accordance with the molecular formula $C_{18}H_{22}O_{10}$, indicating eight degrees of unsaturation. The IR spectrum showed the presence of hydroxy (3390 $cm^{-1}$) and two carbonyl (1729, 1643 $cm^{-1}$) groups. The $^{13}C$ NMR spectrum gave 18 carbon resonances (Table 1). Besides an acetyl signals ($\delta_C$ 20.7, 172.6), and a glucopyranose signals ($\delta_C$ 104.4, 77.8, 75.6, 75.3, 71.5, 64.6), other 10 resonance signals can be ascribable to a tetrasubstituted phenyl ($\delta_C$ 159.3 s, 148.5 s, 135.2 s, 128.7 d, 118.9 d, 116.3 s), two methylenes ($\delta_C$ 33.6 t, 30.3 t), a methine ($\delta_C$ 61.3 d) and a carbonyl ($\delta_C$ 206.5 s), revealing the presence of a trihydroxy-*α*-tetralone.[6] In combination with the $^1H$ NMR spectrum, proton signals due to an tetrasubstituted aromatic ring at $\delta_H$ 6.87 (d, $J$ = 9.0 Hz) and 7.43 (d, $J$ = 9.0 Hz), suggested the 4,5,8-trihydroxy-*α*-tetralone moiety in **1**. The sugar moiety was attached to C-5 of the aglycon on the base of the correlation of H-1′ [$\delta_H$ 4.79 (d, $J$ = 7.5 Hz)] with C-5 ($\delta_C$ 148.5) in the HMBC spectrum (Figure 2). In the $^1H$ NMR spectrum, the coupling constant of the anomeric proton at $\delta_H$

**Figure 2.** Selected 2D NMR correlations of **1**

*To whom correspondence should be addressed. E-mail: ztding@ynu.edu.cn

Springer

**Table 1. NMR spectral data of 1 and 3 in $CD_3OD$ ($^1H$: 500 MHz; $^{13}C$: 125 MHz)**

| | 1 | | 3 | |
|---|---|---|---|---|
| pos. | $\delta_H$ (*J* in Hz) | $\delta_C$ | $\delta_H$ (*J* in Hz) | $\delta_C$ |
| 1 | | 206.5, C | | 206.5, C |
| 2a | 3.06, m | 33.6, $CH_2$ | 3.05, m | 33.6, $CH_2$ |
| 2b | 2.53, m | | 2.50, m | |
| 3a | 2.23, m | 30.3, $CH_2$ | 2.23, m | 30.2, $CH_2$ |
| 3b | 2.20, m | | 2.20, m | |
| 4 | 5.35, t (2.8) | 61.3, CH | 5.35, t (3.0) | 61.3, CH |
| 5 | | 148.5, C | | 148.9, C |
| 6 | 7.43, d (9.0) | 128.7, CH | 7.51, d (9.2) | 128.5, CH |
| 7 | 6.87, d (9.0) | 118.9, CH | 6.86, d (9.2) | 119.0, CH |
| 8 | | 159.3, C | | 159.1, C |
| 9 | | 116.3, C | | 116.2, C |
| 10 | | 135.2, C | | 134.7, C |
| 1′ | 4.79, d (7.5) | 104.4, CH | 4.78, d (7.6) | 104.5, CH |
| 2′ | 3.50, m | 75.3, CH | 3.52, m | 75.4, CH |
| 3′ | 3.44, t (9.0) | 77.8, CH | 3.47, m | 77.9, CH |
| 4′ | 3.35, m | 71.5, CH | 3.40, m | 71.3, CH |
| 5′ | 3.57, m | 75.6, CH | 3.39, m | 78.3, CH |
| 6′a | 4.40, dd (2.0, 11.5) | 64.6, $CH_2$ | 3.72, m | 62.5, $CH_2$ |
| 6′b | 4.24, dd (6.0, 11.5) | | 3.90, m | |
| OAc | 2.03, s | 20.7, $CH_3$ | | |
| | | 172.6, C | | |

4.79 (d, $J$ = 7.5 Hz) indicated that the sugar moiety was a $\beta$-glucopyranose, which was further determined to be $\beta$-D-glucopyranose as judged from the specific rotation ($[\alpha]^{20}_{D}$ + 35.3, *c* 1.0, $H_2O$) obtained on acid hydrolysis of **1**.[7] In addition, correlation from H-6′ ($\delta_H$ 4.40, 4.24) to carbonyl carbon ($\delta_C$ 172.6) of the acetyl group was also observed in the HMBC spectrum, indicating that the acetyl group was linked to C-6′. Therefore, the planar structure of **1** was elucidated and was designated as 6'-*O*-acetyl-juglanoside E.

C-4 of **1** was assumed to be *S* because of co-occurrence of other (4*S*)-4,5,8-trihydroxy-$\alpha$-tetralone isolated from *Juglans*.[6,8,9] Moreover, (4*R*)-4,5,8-trihydroxy-$\alpha$-tetralone from microbial metabolites has not been reported in plants yet.[10]

Compound **2** was isolated as white amorphous powder with mp 106.1–108.2 °C. Its molecular formula $C_{23}H_{34}O_{11}$, was derived from HRESIMS ($[M + Na]^+$ 509.1988, calcd. for $C_{23}H_{34}O_{11}Na$, 509.1998). The IR spectrum displayed the absorption bands for OH (3398 $cm^{-1}$), C=O (1708 $cm^{-1}$), C=C (1601 $cm^{-1}$), and C-O-C (1071, 1037 $cm^{-1}$) functionalities. NMR spectra of **2** (Table 2) revealed the presence of one $\beta$-glucosyl moiety [$\delta_H$ 5.52 (1H, d, $J$ = 8.2 Hz); $\delta_C$ 95.3 d, 77.8 d, 76.1 d, 73.9 d, 71.2 d, 64.5 t] and an acetyl group [$\delta_H$ 2.07 (3H, s); $\delta_C$ 172.8 s, 20.7 q]. Acid hydrolysis of **1** and comparison of the specific rotation of the acid hydrolysate ($[\alpha]^{20}_{D}$ + 36.5, *c* 1.0, $H_2O$) with an authentic standard of D-glucose established the sugar as D-glucose.[7] The remaining 15 carbons were ascribable for three methyls, three methylenes, four methines, four quaternary carbons, and one carbonyl group, suggesting the presence of a dihydrophaseic acid moiety in **2**.[11] HMBC correlation of H-1′ [$\delta_H$ 5.52 (d, $J$ = 8.2 Hz)] with C-1 ($\delta_C$ 165.8) established the $\beta$-D-glucopyranosyl unit located at C-1. The HMBC correlation of H-6′ [$\delta_H$ 4.23 (dd, $J$ = 5.5, 12.0 Hz), 4.39 (d, $J$ = 12.0 Hz)] with the carbonyl signal of acetyl group at $\delta_C$ 172.8 revealed that the acetyl group could be located at C-6′ in the glucosyl moiety. Thus, the planar structure of **2** was elucidated as shown.

The cyclohexane ring in **2** appears to be a chair conformation based on the literatures[12,13], the spin-spin splitting patterns, and the coupling constants of H-8b (t, $J$ = 13.0 Hz), H-9 (tt, $J$ = 13.0, 7.0 Hz), and H-10b (t, $J$ = 13.0 Hz).[14] On the beta side of the cyclohexane plane, H-8b ($\delta_H$ 1.75) and H-10b ($\delta_H$ 1.67) have to be axial because of the NOE correlations of H-5 [$\delta_H$ 6.63 (d, $J$ = 16.0 Hz)] with H-8b and H-5 with H-10b (Figure 3),[12] while the absence of NOE correlations of H-9 with H-8b and H-10b indicated H-9 ($\delta_H$ 4.12) was also in axial on the alpha side of the ring. The presence of a clear NOE correlation between H-9 and H-12a [$\delta_H$ 3.74 (d, $J$ = 7.5 Hz)] which was not observed in *epi*-dihydrophaseic acid derivatives[12] supported above conclusion as well. In addition, oxymethylene carbon C-12 has to be in axial orientation on the alpha side of the ring (Figure 3), as a stereo assumption for a successful ether ring formation in system **2**.[12] OH-6 and the oxymethylene bridge should be on the same side of the ring system due to almost superimposed NMR signals of the two oxymethylene protons (H-12) (Table 2).[13,15] Further NMR data comparison between **2** and both dihydrophaseic acid and *epi*-dihydrophaseic acid could give another evidence.[15] The $^1H$ NMR data of **2** was in good accordance to that of dihydrophaseic acid (**4**) (Table 2), but exhibited some differences from that of *epi*-dihydrophaseic acid, especially for H-5. In **2**, this signal occur 0.23 ppm further downfield, which was accounted for OH-9 can approach of the side chain.[13] Thus, OH-9 and the C-5 side chain have to be in the same beta side of the cyclohexane ring. At last, the configurations of the double bonds in **2** were determined via 1D and 2D NMR. The (*E*) configuration of C(4)=C(5) bond was assigned based on the coupling constants,[13,16] and a ROESY cross-peak (Figure 3) between H-2 and Me-15 showed the configuration of C(2)=C(3) bond is (*Z*). So far, the relative configuration of **2** was confirmed and named as dihydrophaseic acid 1-*O*-(6-*O*-acetyl)-glucopyranoside.

**Figure 3.** Selected 2D NMR correlations of **2**

## Experimental Section

**General Experimental Procedures.** Melting point was measured on a Dresden HMK micromelting point apparatus and was uncorrected. The high resolution 1D and 2D NMR spectra (HMQC, HMBC, $^1H$-$^1H$ COSY and ROESY) were performed using Bruker DRX-500 MHz spectrometers. All chemical shifts ($\delta$) are given in ppm, and TMS was used as an internal standard. MS spectra were measured on a VG Auto Spec-3000 mass spectrometer and Agilent G3250AA LC/MSD TOF spectrometer. IR was measured on a Nicolet FTIR 5DX spectrophotometer with KBr pellets. Specific rotation was obtained on a JASCO P-1020 digital polarimeter. Silica gel (200–300 mesh) was the product of the Qingdao Marine Chemical Ltd. Sephadex LH-20 was purchased from

**Table 2. NMR spectral data of 2 and 4 in $CD_3OD$ ($^1H$: 500 MHz; $^{13}C$: 125 MHz)**

| | **2** | | **4** | |
|---|---|---|---|---|
| pos. | $\delta_H$ (*J* in Hz) | $\delta_C$ | $\delta_H$ (*J* in Hz) | $\delta_C$ |
| 1 | | 165.8, C | | 169.6, C |
| 2 | 5.86, s | 117.7, CH | 5.76, s | 119.2, CH |
| 3 | | 154.0, C | | 151.5, C |
| 4 | 8.05, d (16.0) | 131.7, CH | 7.89, d (16.0) | 131.8, CH |
| 5 | 6.63, d (16.0) | 136.6, CH | 6.52,d (16.0) | 135.2, CH |
| 6 | | 83.3, C | | 83.2, C |
| 7 | | 87.8, C | | 87.8, C |
| 8a | 2.05, overlap | 46.0, $CH_2$ | 2.03, m | 46.0, $CH_2$ |
| 8b | 1.75, t (13.0) | | 1.73, m | |
| 9 | 4.12, tt (13.0, 7.0) | 66.0, CH | 4.11, m | 66.0, CH |
| 10a | 1.85, dd (13.0, 7.0) | 44.5, $CH_2$ | 1.83, m | 44.5, $CH_2$ |
| 10b | 1.67, t (13.0) | | 1.67, m | |
| 11 | | 49.4, C | | 49.4, C |
| 12a | 3.74, d (7.5) | 77.3, $CH_2$ | 3.70, d (8.8) | 77.3, $CH_2$ |
| 12b | 3.83, d (7.5) | | 3.80, d (8.8) | |
| 13 | 0.96, s | 16.3, $CH_3$ | 0.92, s | 16.4, $CH_3$ |
| 14 | 1.18, s | 19.7, $CH_3$ | 1.14, s | 19.7, $CH_3$ |
| 15 | 2.15, s | 21.3, $CH_3$ | 2.08, s | 21.3, $CH_3$ |
| 1′ | 5.52, d (8.0) | 95.3, CH | | |
| 2′ | 3.40, m | 73.9, CH | | |
| 3′ | 3.46, t (9.0) | 77.8, CH | | |
| 4′ | 3.39, m | 71.2, CH | | |
| 5′ | 3.62, m | 76.1, CH | | |
| 6′a | 4.23, d (12.0) | 64.5, $CH_2$ | | |
| 6′b | 4.39, dd (5.5, 12.0) | | | |
| OAc | 2.07 s | 20.7, $CH_3$ | | |
| | | 172.8, C | | |

Amersham Biosciences. RP-18 was purchased from Merck (Merck, Darmstadt, Germany). A Waters 600 series instrument equipped with Waters Spherisorb® ODS2 column (5 $\mu$m, 4.6 mm × 250 mm) was used for high-performance liquid chromatography (HPLC) analysis, and a semi-preparative Wters Spherisorb® S10 ODS2 column (5 $\mu$m, 20 mm × 250 mm) was used for the sample preparation.

**Plant Materal.** The pellicle of *Juglans regia* was collected in Kunming, Yunnan Province, China, in August 2008, and authenticated by Prof. Shu-Gang Lu from School of Life Science, Yunnan University. A voucher specimen (No. LCS-02) has been deposited in the Key Laboratory of Medicinal Chemistry for Nature Resource of Yunnan University.

**Extraction and Isolation.** The dried pellicle of *Juglans regia* (1.8 kg) was powdered and extracted three times with 80% aqueous acetone at room temperature. The extracts were combined, and evaporated under reduced pressure. The crude residue was suspended in $H_2O$, and partitioned with petroleum ether, EtOAc, and *n*-butanol successively. The *n*-butanol fraction (140 g) was subjected to silica gel column eluted gradually with $CHCl_3$/MeOH (200:1–1:1) to yield eight fractions (Fr. C1–C8). Fr. C3 (6.3 g) was chromatographed on a silica gel eluted with EtOAc/MeOH (20:1) and then purified on Sephadex LH-20 (MeOH), yielding **4** (9 mg). Fr. C5 (1.2 g) was subjected to MCI gel, Sephadex LH-20 and semi-preparative HPLC successively to yield **1** (11 mg), **2** (9 mg) and **3** (8 mg).

**6'-*O*-acetyl-juglanoside E (1):** white powder; mp 84.5–87.2 °C; $[\alpha]^{20}_D$ – 9.02 (*c* 1.28, MeOH); IR (KBr) $\nu_{max}$: 3390, 2921, 1729, 1643, 1465, 1239, 1073 $cm^{-1}$; $^1H$ and $^{13}C$ NMR data see Table 1; HRESIMS *m*/*z*: 421.1112 $[M + Na]^+$ (calcd for $C_{18}H_{22}O_{10}Na$ $[M + Na]^+$, 421.1110).

**Dihydrophaseic acid 1-*O*-(6-*O*-acetyl)-glucopyranoside (2):** white amorphous powder; mp 106.1–108.2 °C; $[\alpha]^{20}_D$ – 5.33 (*c* 1.90, MeOH); IR (KBr) $\nu_{max}$**:** 3398, 2931, 1708, 1601, 1236, 1071, 1037 $cm^{-1}$; $^1H$ and $^{13}C$ NMR data see Table 2; HRESIMS *m*/*z*: 509.1988 $[M + Na]^+$ (calcd for $C_{23}H_{34}O_{11}Na$ $[M + Na]^+$, 509.1998).

**Acidic Hydrolysis of 1 and 2.** 5 mg of **1** and **2** was added 3 mL of 2 N HCl, and the reaction mixture was heated at 90 °C on a water bath for 2 h. After workup, the reaction mixture was extracted three times with 2 mL of EtOAc. The aqueous phase was concentrated under reduced pressure to give a residue. Comparison of the optical rotations for each hydrolyzed product of **1** ($[\alpha]^{20}_D$ + 35.3, *c* 1.0, $H_2O$) and **2** ($[\alpha]^{20}_D$ + 36.5, *c* 1.0, $H_2O$) with an authentic standard confirmed that the glucose was D-glucose.

## Electronic Supplementary Material

Supplementary material is available in the online version of this article at http://dx.doi.org/ 10.1007/s13659-012-0009-0 and is accessible for authorized users.

## Acknowledgments

This work was financially supported by a program for New Century Excellent Talents in University from MOE (No. NCET-08-0925), together with a grant from the Natural Science Foundation of China (21002084), grants from the Natural Science Foundation of the Yunnan Province (Nos. 2008CD066, 2010CD017), an Undergraduates Innovative Experiment Project from MOE (101067320), as well as grants from Key Laboratory of Medicinal Chemistry for Nature Resource, MOE (Nos. 2009102204, 2009102202).

**Open Access** This article is distributed under the terms of the Creative Commons Attribution License which permits any use, distribution, and reproduction in any medium, provided the original author(s) and source are credited.

## Electronic supplementary material

Supplementary material, approximately 7.00 MB.
