# Supplementary material for: Two new glucosides from the pellicle of the walnut (Juglans regia)
Source: Nat Prod Bioprospect. 2012 Jun 22;2(4):150–3. doi: 10.1007/s13659-012-0009-0 (PMC4131628; doi:10.1007/s13659-012-0009-0)

Electronic Supplementary Material

Two new glucosides from the pellicle of the walnut (*Juglans regia*)

Le CAI, Chuan-Shui LIU, Xiao-Wei FU, Xiao-Jing SHEN, Tian-Peng YIN, Ya-Bin YANG, and Zhong-Tao DING*

Key Laboratory of Medicinal Chemistry for Nature Resource, Ministry of Education, School of Chemical Science and Technology, Yunnan University, Kunming 650091, Yunnnan, China

Received 5 February 2012; Accepted 21 March 2012

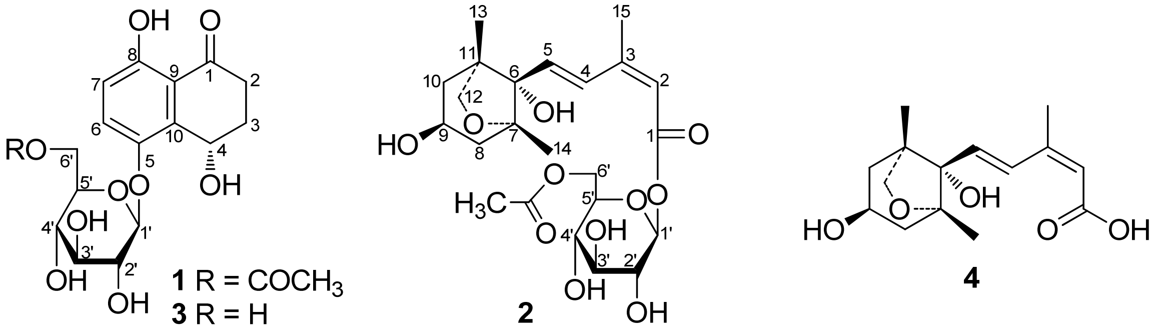

Structures of compounds **1**–**4**

*To whom correspondence should be addressed. E-mail: ztding@ynu.edu.cn

Supporting Information Contents

1. Optical rotation data of **1**……2
2. IR data of **1**……3
3. ^{1}H NMR data of **1**……4
4. ^{13}C NMR data of **1**……5
5. ^{1}H-^{1}H COSY data of **1**……6
6. HSQC data of **1**……7
7. HMBC data of **1**……8
8. NOESY data of **1**……9
9. ESIMS data of **1**……10
10. HRESIMS data of **1**……11
11. Optical rotation data of **2**……14
12. IR data of **2**……15
13. ^{1}H NMR data of **2**……16
14. ^{13}C NMR data of **2**……17
15. ^{1}H-^{1}H COSY data of **2**……18
16. HSQC data of **2**……19
17. HMBC data of **2**……20
18. NOESY data of **2**……21
19. ESIMS data of **2**……22
20. HRESIMS data of **2**……23

Optical rotation data of **1**

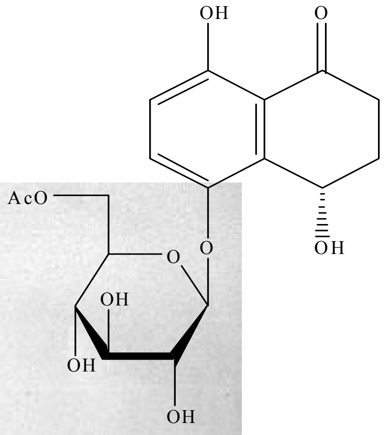

Optical rotation measurement

Model : P-1020 (A060460638)

No.	Sample	Mode	Data	Monitor<br>Blank	Temp.<br>Cell<br>Temp Point	Date<br>Comment<br>Sample Name	Light<br>Filter<br>Operator	Cycle Time<br>Integ Time
No.1	6 (1/3)	Sp.Rot	-9.1250	-0.0584<br>0.0000	16.8<br>50.00<br>Cell	Mon Nov 29 13:54:01 2010<br>0.01280g/mlMeOH<br>35#	Na<br>589nm	2 sec<br>10 sec
No.2	6 (2/3)	Sp.Rot	-8.7500	-0.0560<br>0.0000	16.8<br>50.00<br>Cell	Mon Nov 29 13:54:14 2010<br>0.01280g/mlMeOH<br>35#	Na<br>589nm	2 sec<br>10 sec
No.3	6 (3/3)	Sp.Rot	-9.1880	-0.0588<br>0.0000	16.8<br>50.00<br>Cell	Mon Nov 29 13:54:28 2010<br>0.01280g/mlMeOH<br>35#	Na<br>589nm	2 sec<br>10 sec

-9.0208°

IR data of **1**

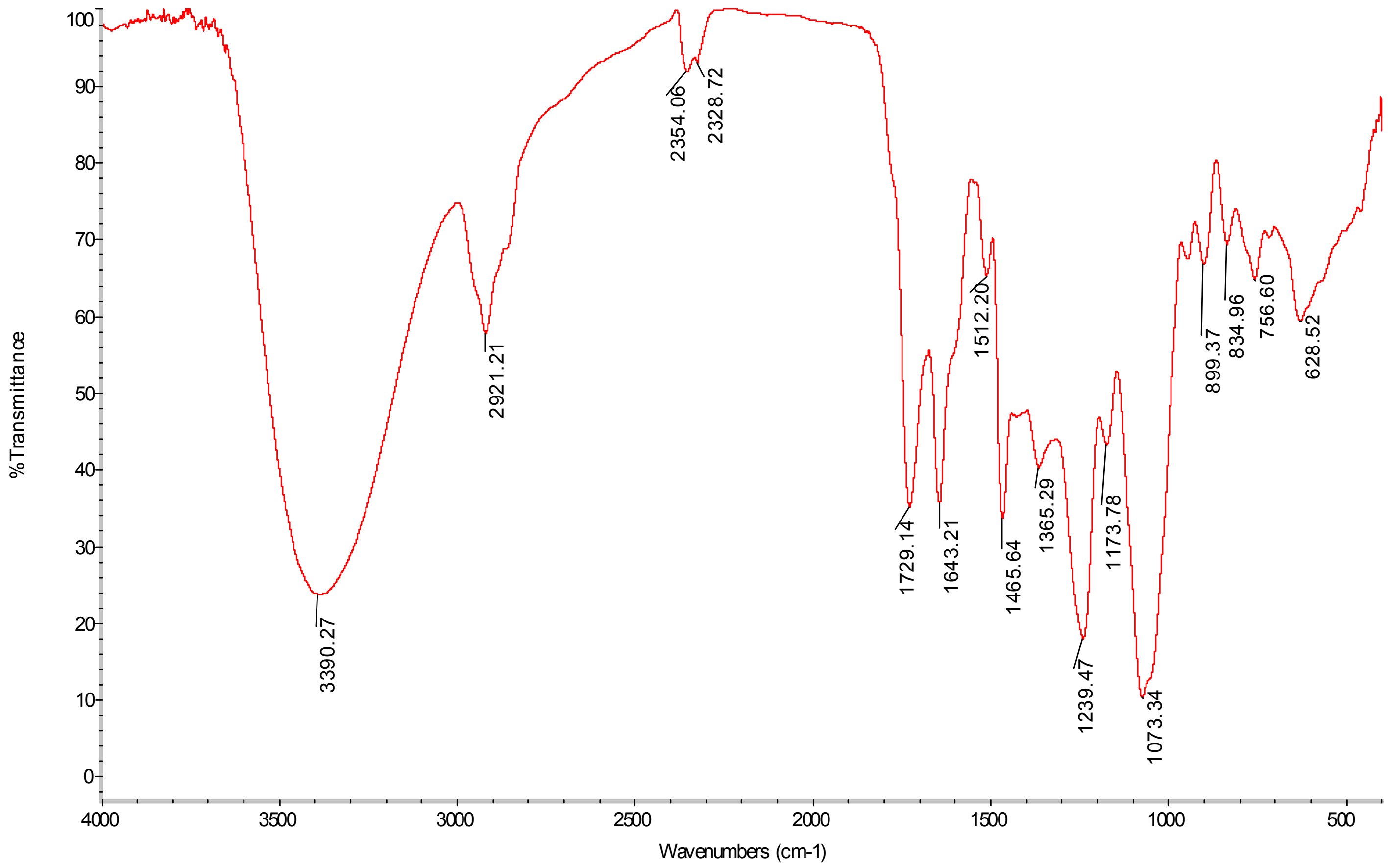

%Transmittance
Wavenumbers (cm-1)
3390.27
2921.21
2354.06
2328.72
1729.14
1643.21
1512.20
1465.64
1365.29
1239.47
1173.78
1073.34
899.37
834.96
756.60
628.52

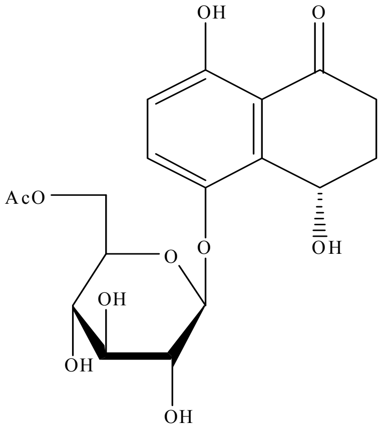

OH
O
AcO
O
O
OH
OH
OH
OH

^{1}H NMR data of **1** (CD_3OD, 500 MHz)

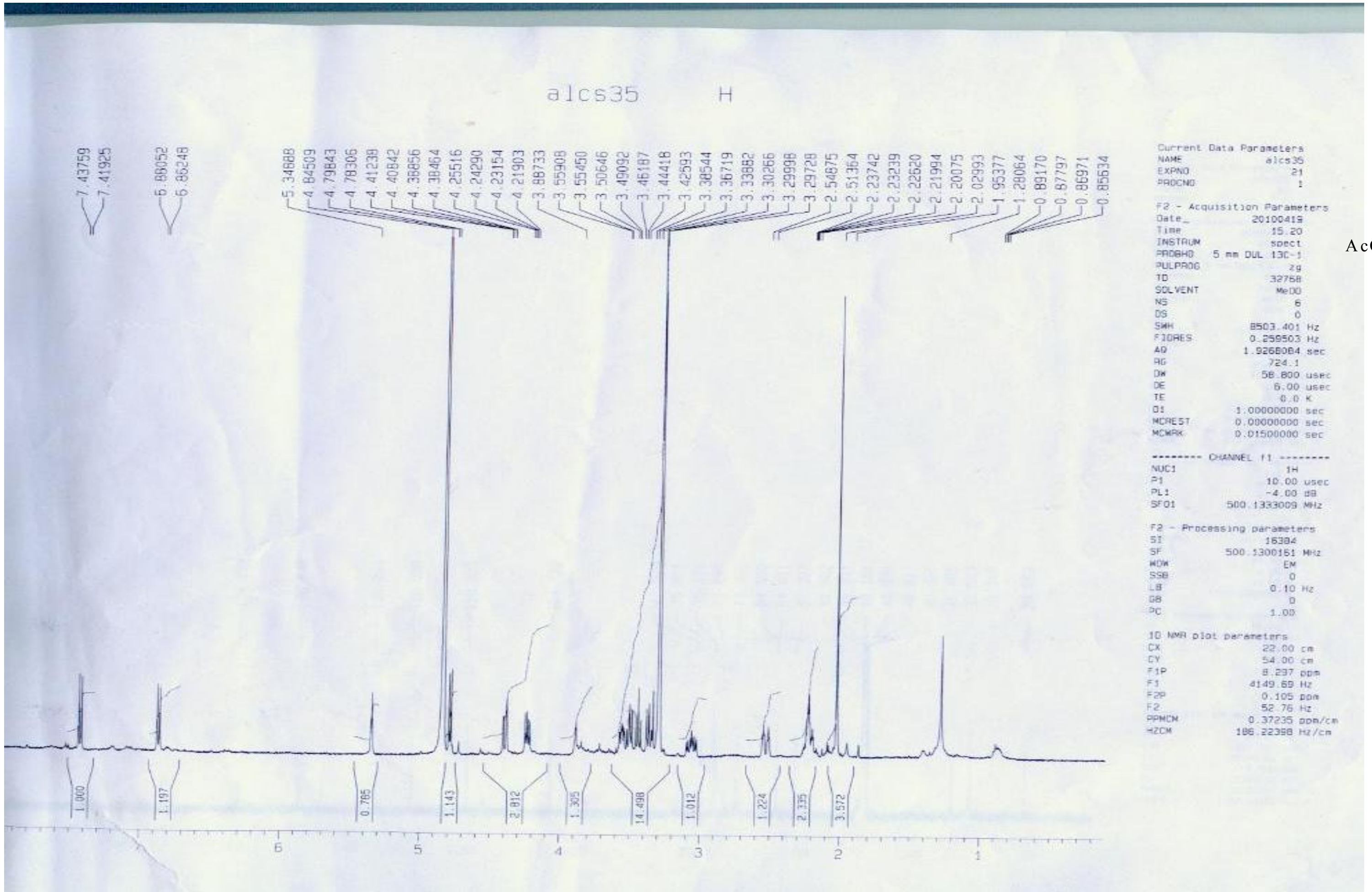

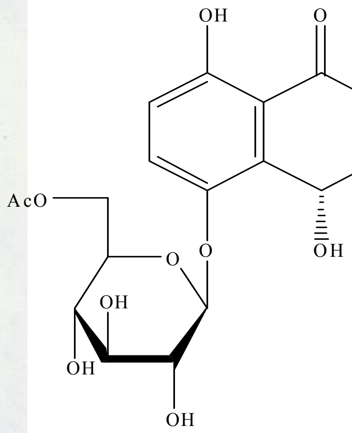

^{13}C NMR data of **1** (CD_3OD, 125 MHz)

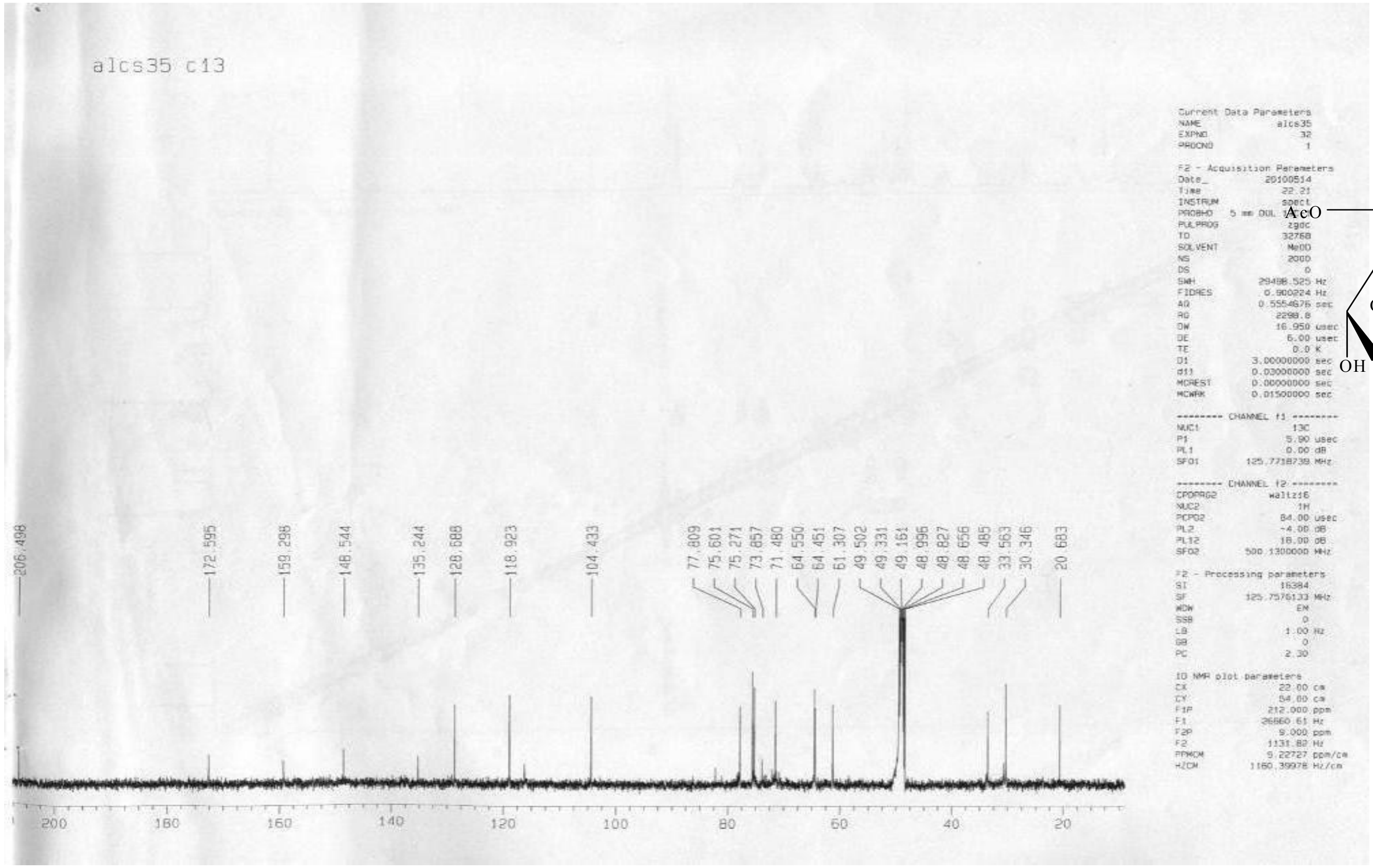

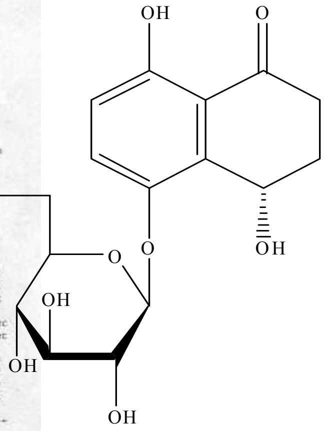

^1H-^1H COSY data of **1**

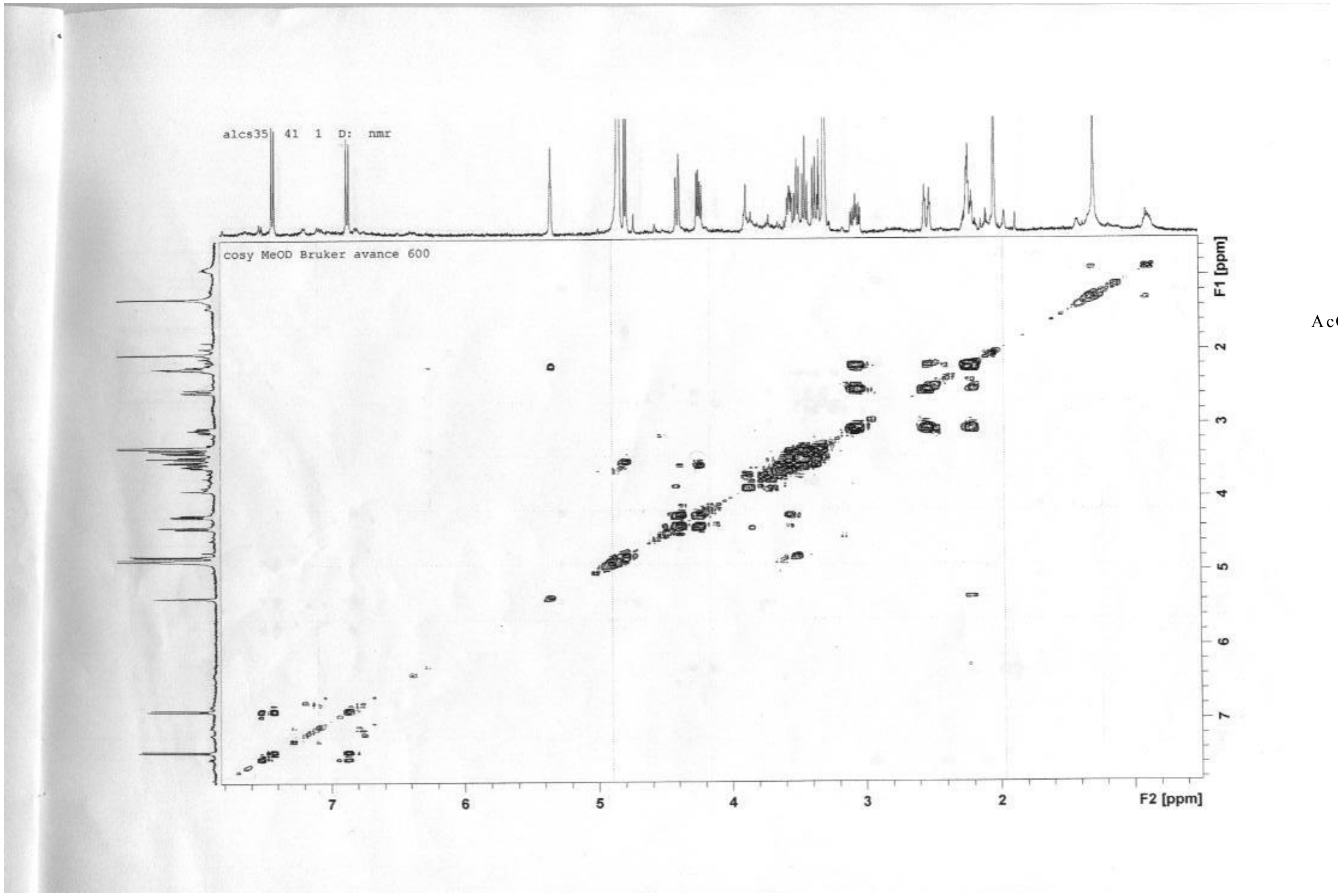

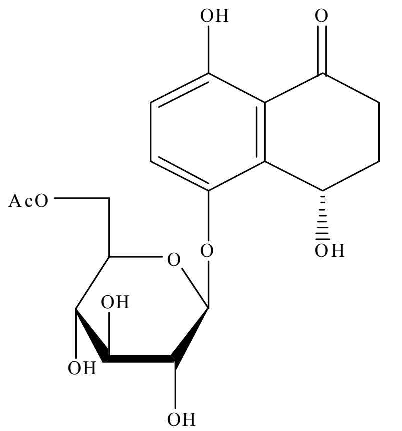

HSQC data of **1**

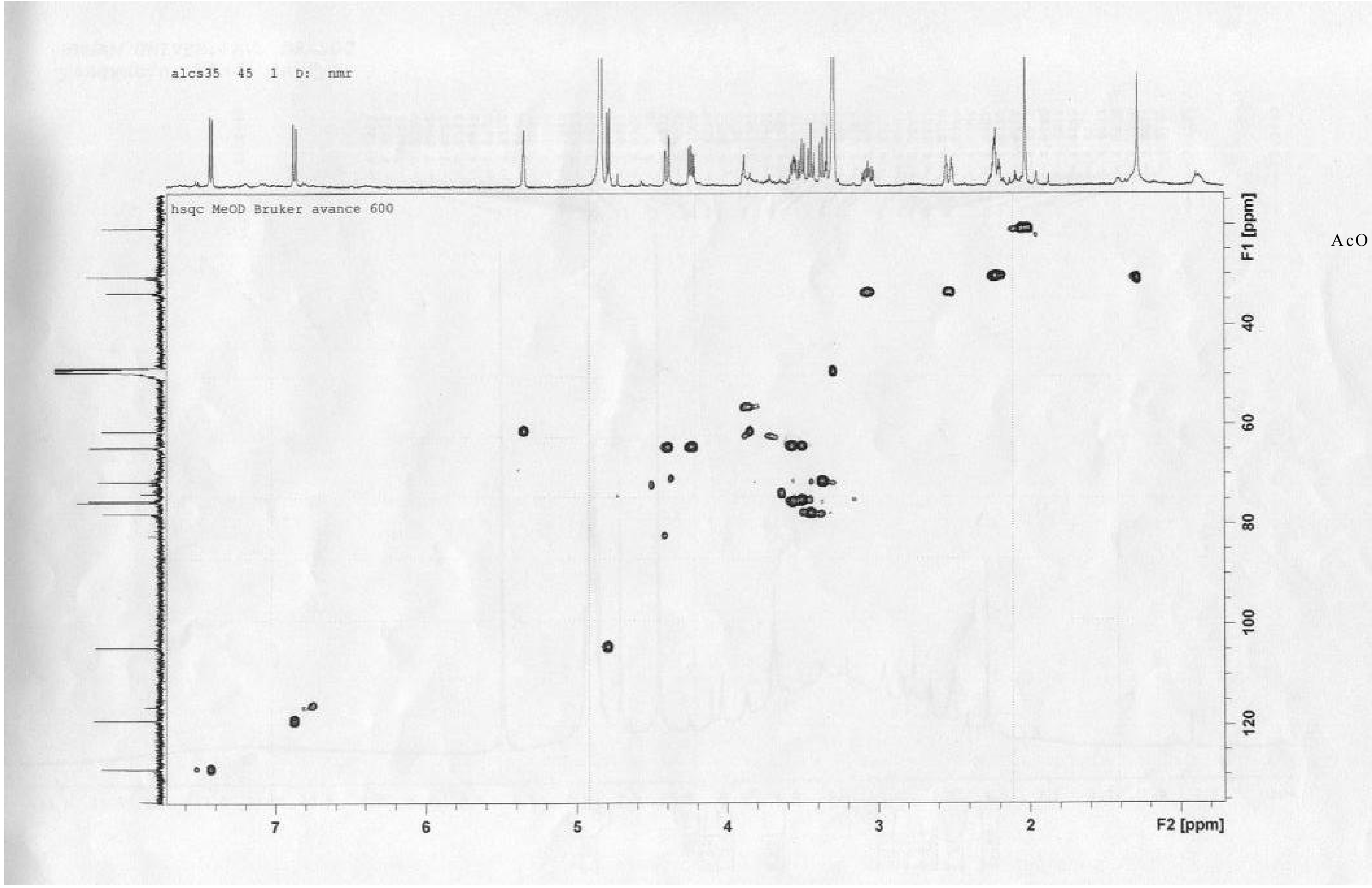

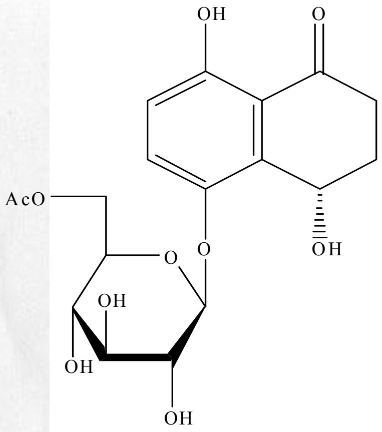

HMBC data of **1**

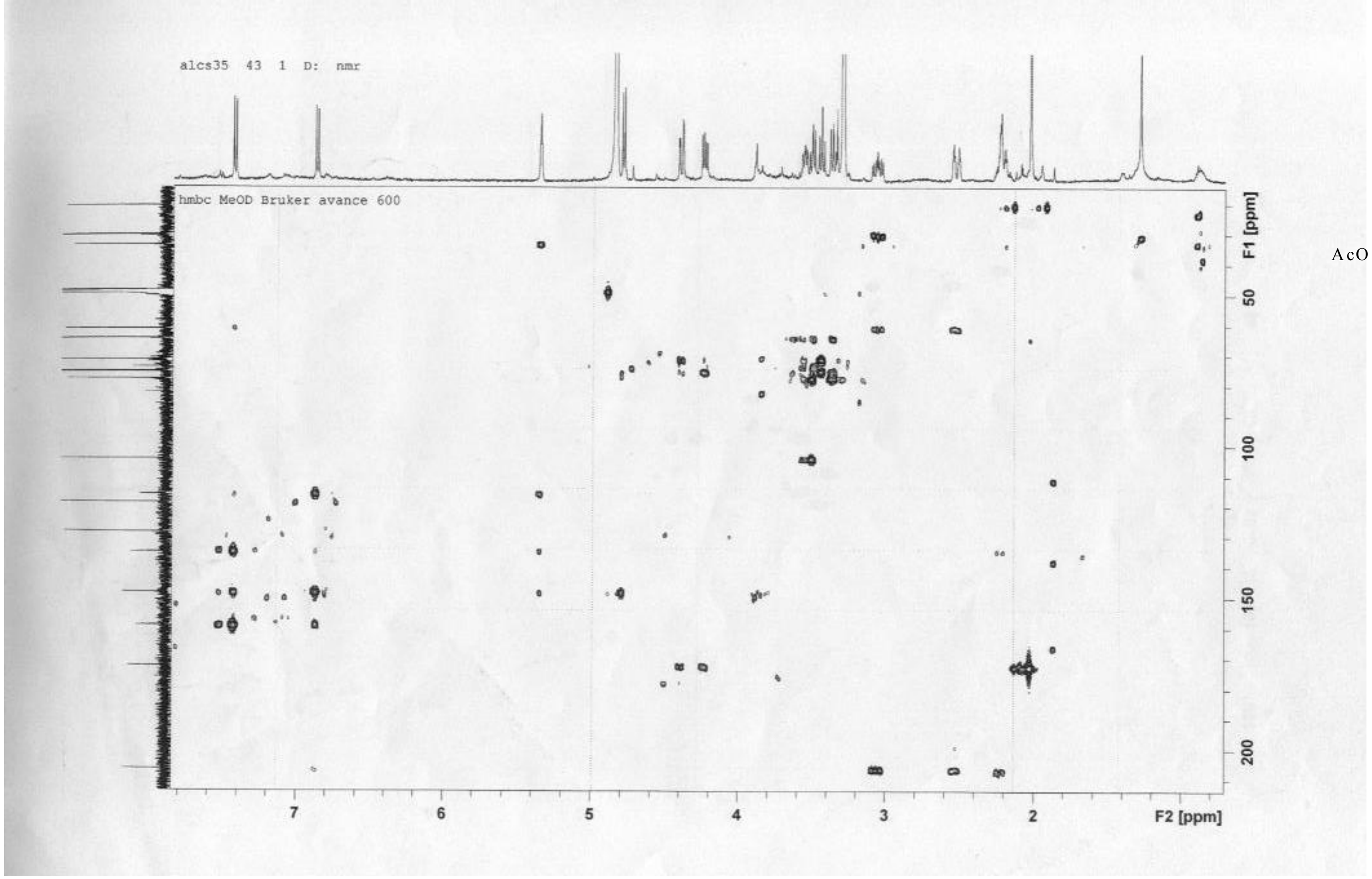

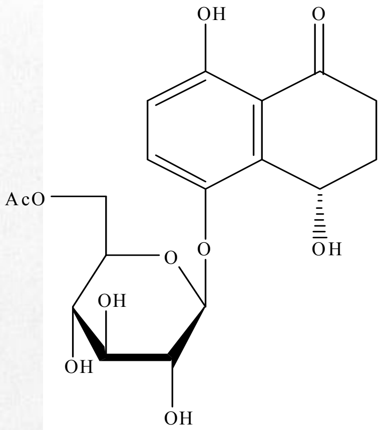

ROESY data of **1**

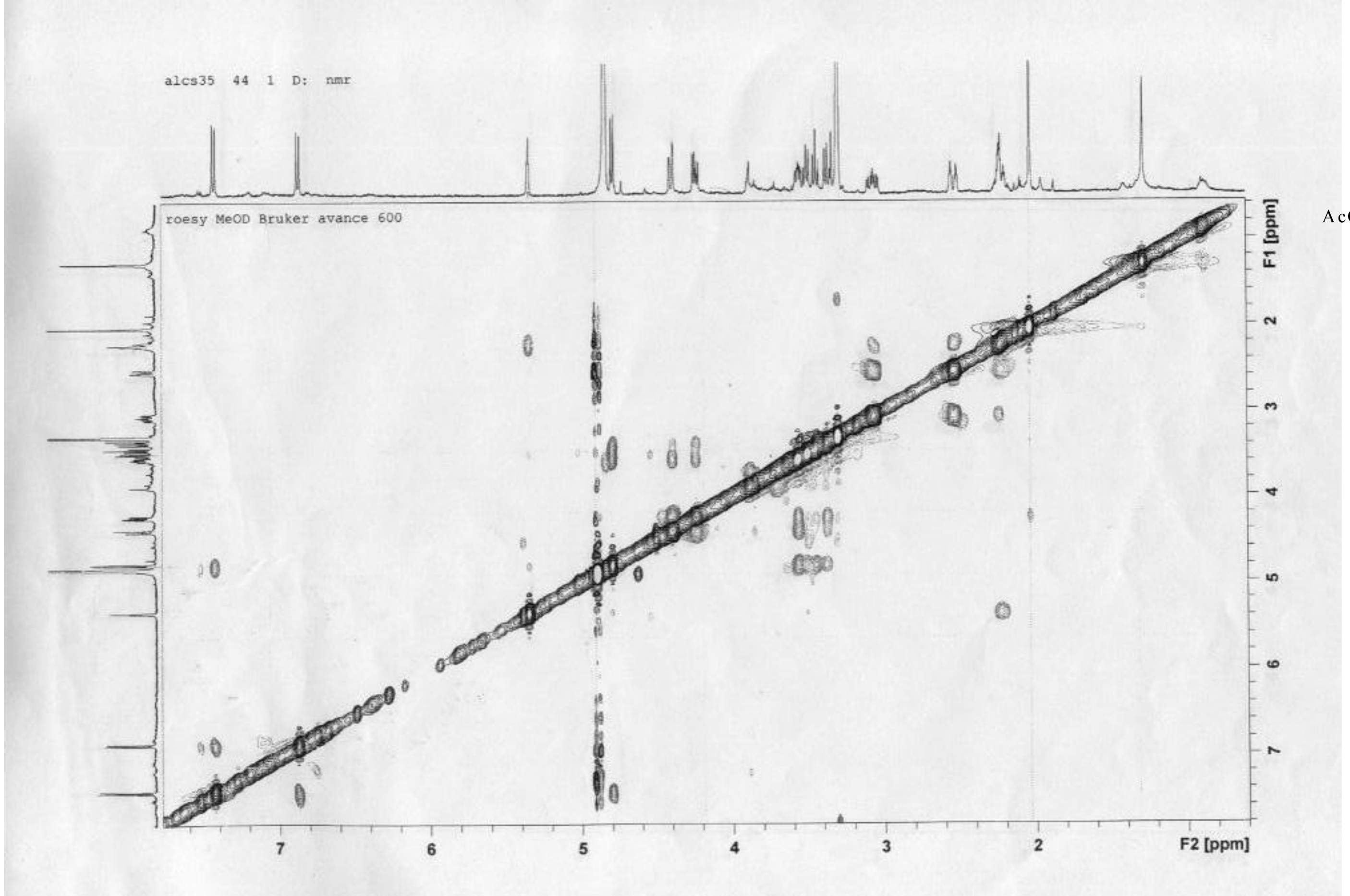

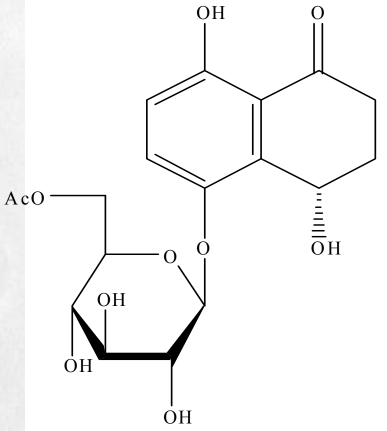

ESIMS data of **1**

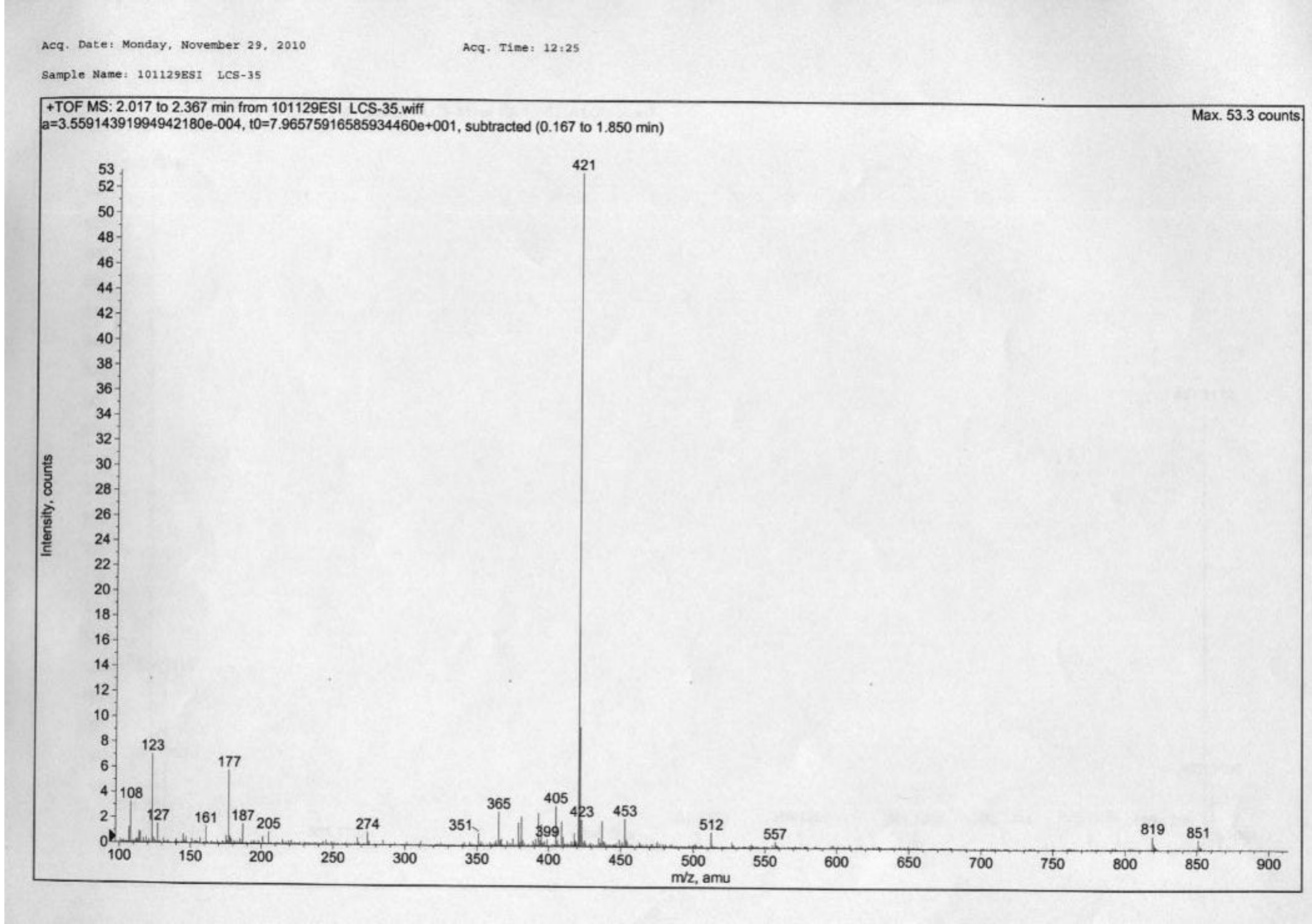

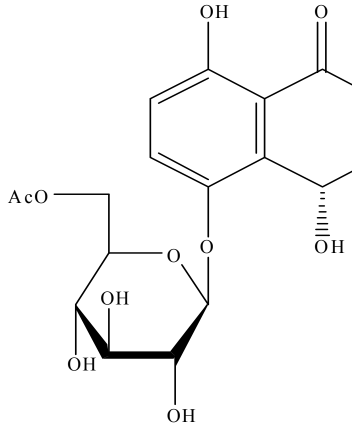

HRESIMS data of **1**

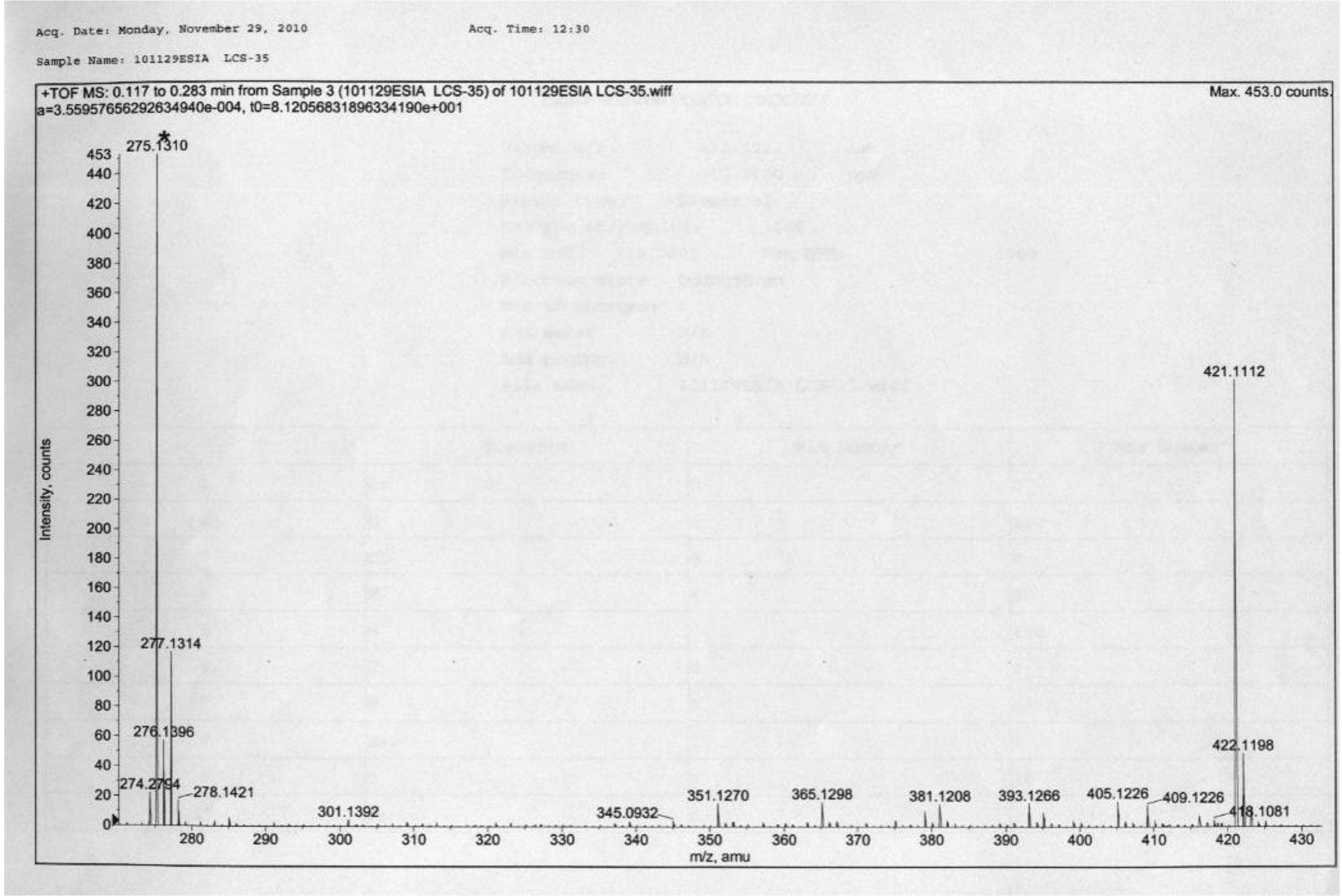

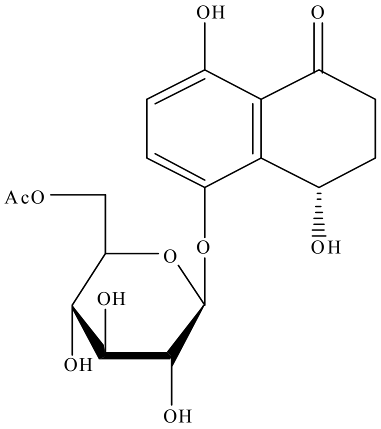

HRESIMS data of **1**

Acq. Date: Monday, November 29, 2010 Acq. Time: 12:30

Sample Name: 101129ESIA LCS-35

Elemental composition calculator

Target m/z: +421.1112 amu
Tolerance: +10.0000 ppm
Result type: Elemental
Max num of results: 1000
Min DBE: -10.0000 Max DBE: +60.0000
Electron state: OddAndEven
Num of charges: 0
Add water: N/A
Add proton: N/A
File Name: 101129ESIA LCS-35.wiff

	Elements	Min Number	Max Number
1	Br	0	0
2	C	0	200
3	Cl	0	0
4	F	0	0
5	H	0	400
6	K	0	0
7	N	0	0
8	Na	1	1
9	O	7	10
10	Pt	0	0

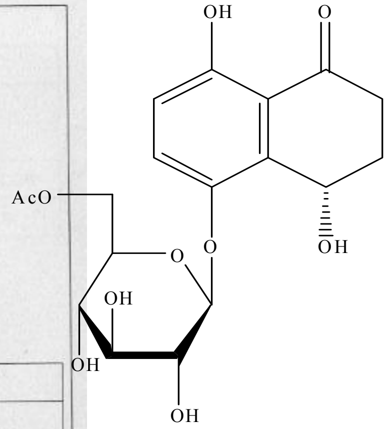

HRESIMS data of **1**

Acq. Date: Monday, November 29, 2010 Acq. Time: 12:30

Sample Name: 101129ESIA LCS-35

	Elements	Min Number	Max Number
11	S	0	0
12	Si	0	0

	Formula	Calculated m/z (amu)	mDa Error	PPM Error	DBE
1	C18 H22 O10 Na	421.1110	0.1330	0.3158	7.5

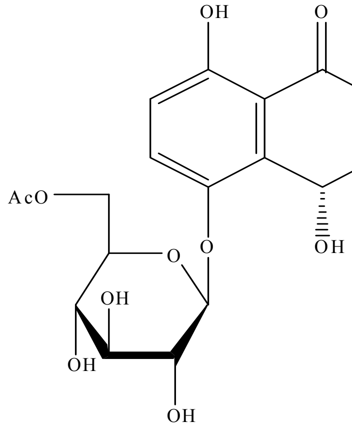

Optical rotation data of **2**

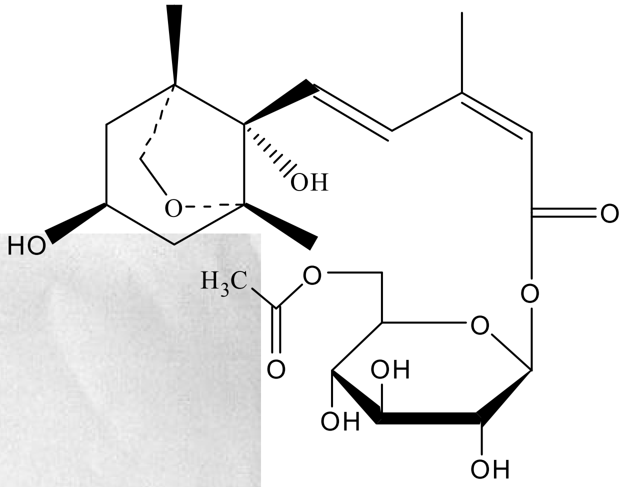

Optical rotation measurement

Model : P-1020 (A060460638)

No.	Sample	Mode	Data	Monitor<br>Blank	Temp.<br>Cell<br>Temp Point	Date<br>Comment<br>Sample Name	Light<br>Filter<br>Operator	Cycle Time<br>Integ Time
No.1	8 (1/3)	Sp.Rot	-5.4640	-0.0265<br>0.0000	17.4<br>50.00<br>Cell	Mon Nov 29 14:34:49 2010<br>0.00970g/mlMeOH<br>49#	Na<br>589nm	2 sec<br>10 sec
No.2	8 (2/3)	Sp.Rot	-5.0520	-0.0245<br>0.0000	17.4<br>50.00<br>Cell	Mon Nov 29 14:35:02 2010<br>0.00970g/mlMeOH<br>49#	Na<br>589nm	2 sec<br>10 sec
No.3	8 (3/3)	Sp.Rot	-5.4640	-0.0265<br>0.0000	17.4<br>50.00<br>Cell	Mon Nov 29 14:35:15 2010<br>0.00970g/mlMeOH<br>49#	Na<br>589nm	2 sec<br>10 sec

-5.3265°

IR data of **2**

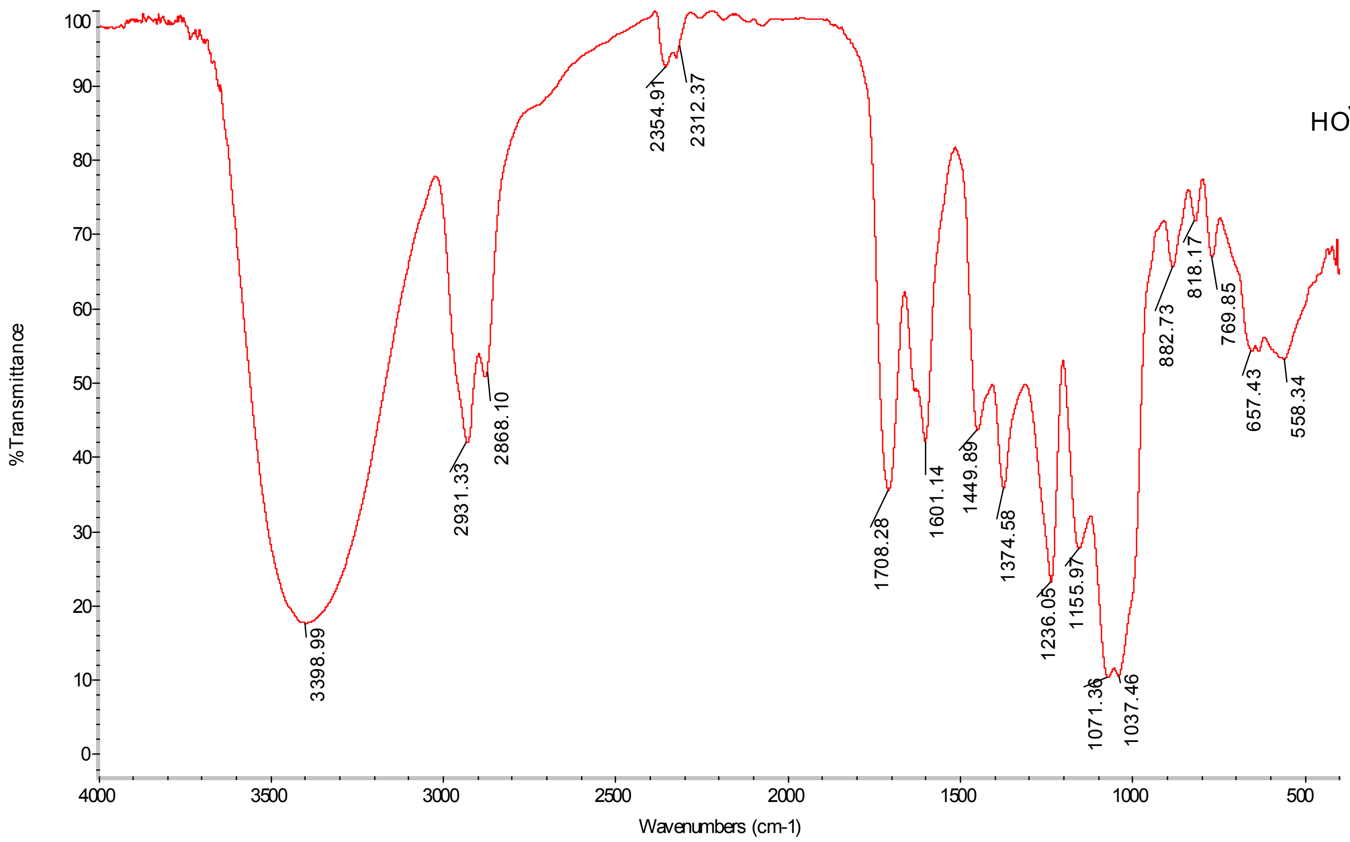
%Transmittance
Wavenumbers (cm-1)
100
90
80
70
60
50
40
30
20
10
0
4000
3500
3000
2500
2000
1500
1000
500
3398.99
2931.33
2868.10
2354.91
2312.37
1708.28
1601.14
1449.89
1374.58
1236.05
1155.97
1071.36
1037.46
882.73
818.17
769.85
657.43
558.34

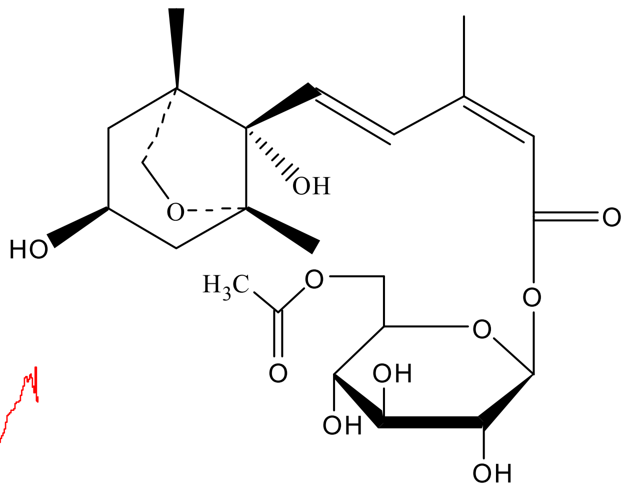
HO
OH
O
O
O
H3C
O
O
O
OH
OH
OH

^{1}H NMR data of 2 (CD_3OD, 500 MHz)

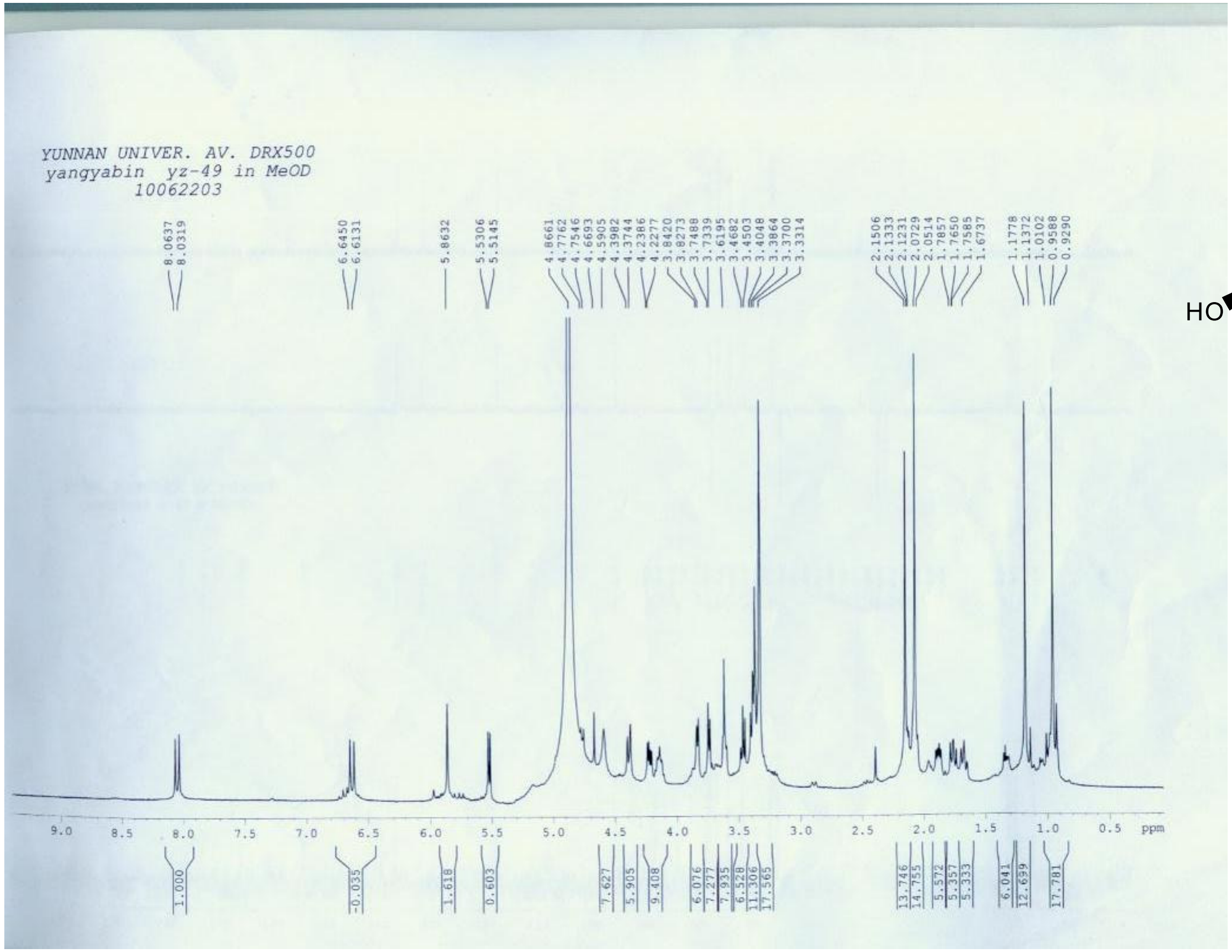

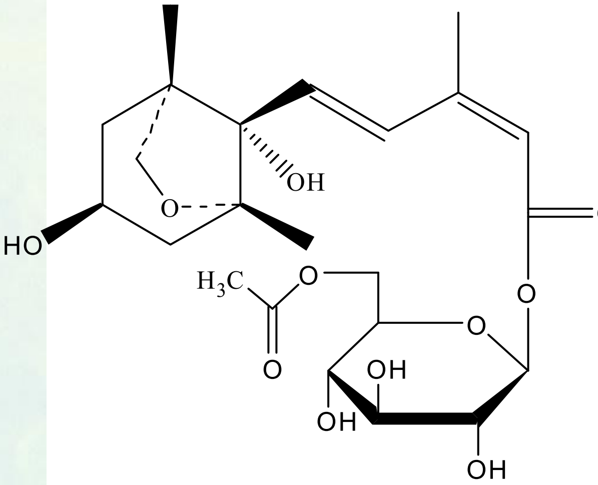

^{13}C NMR data of **2** (CD_3OD, 125 MHz)

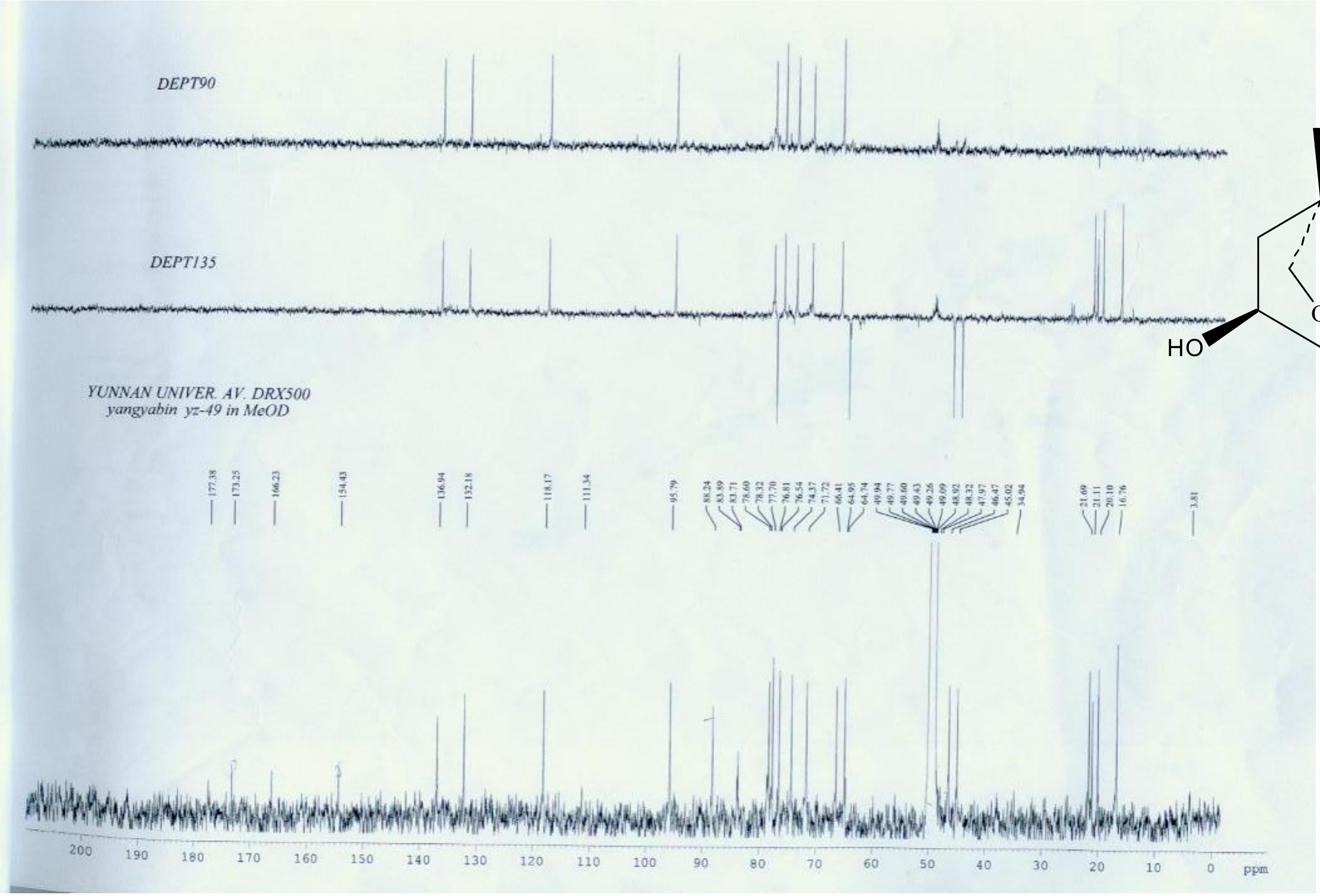

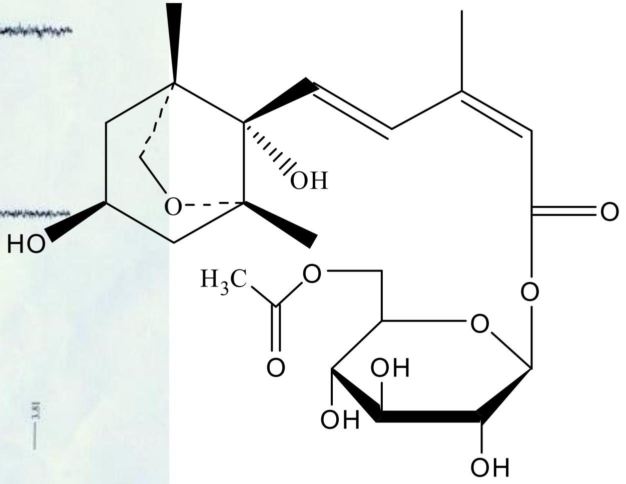

^1H-^1H COSY data of **2**

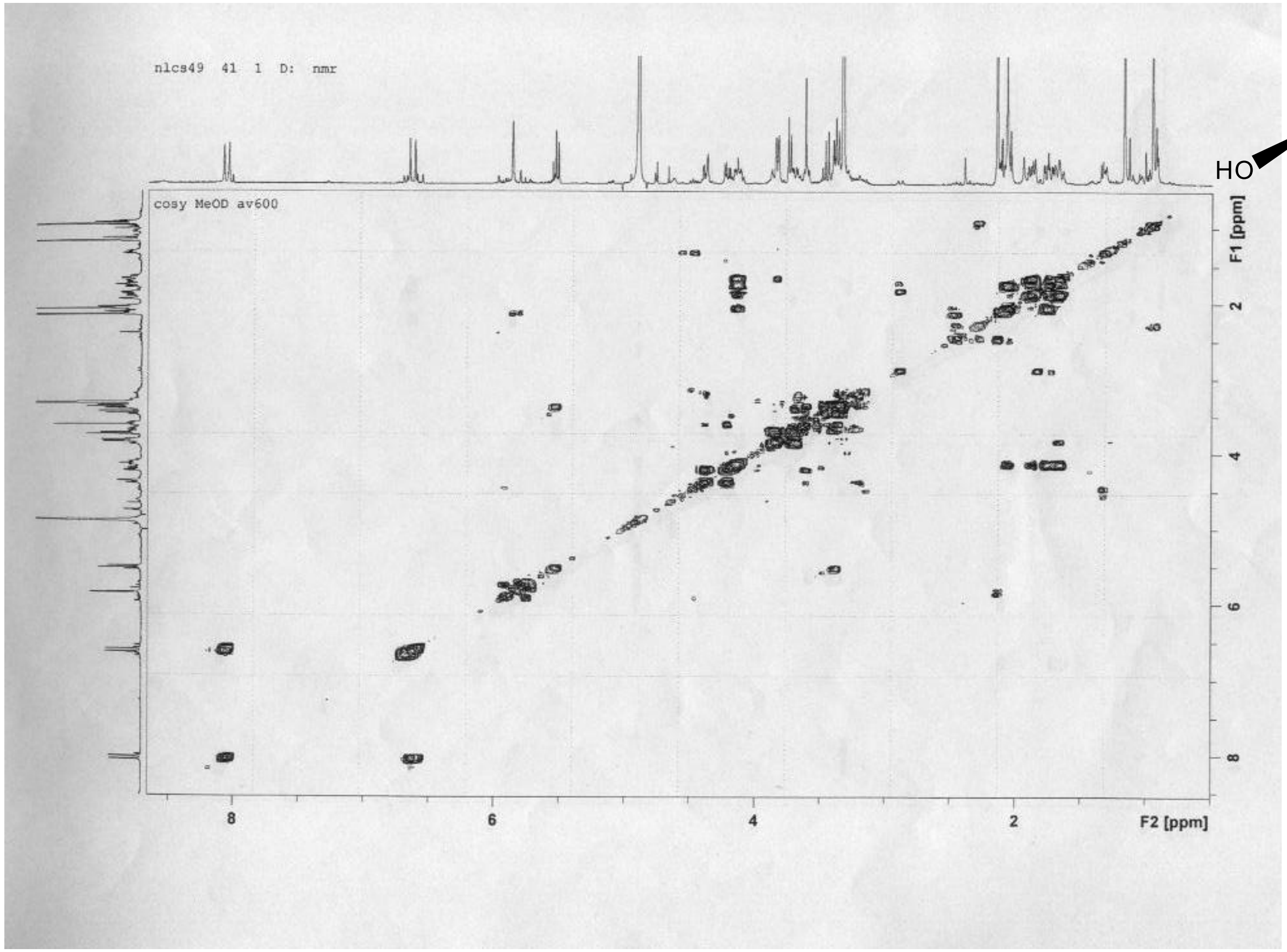

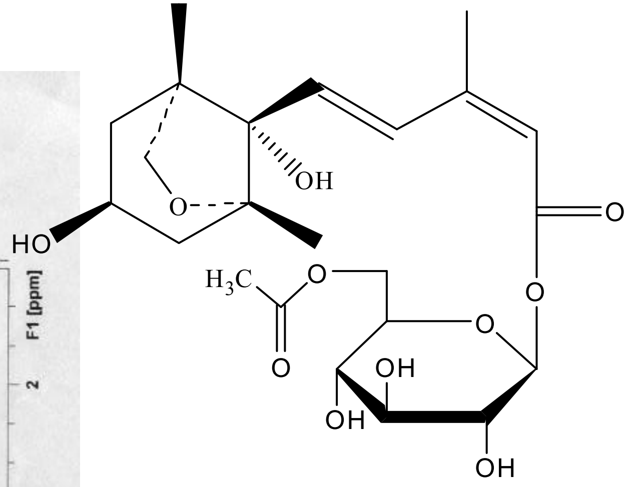

HSQC data of **2**

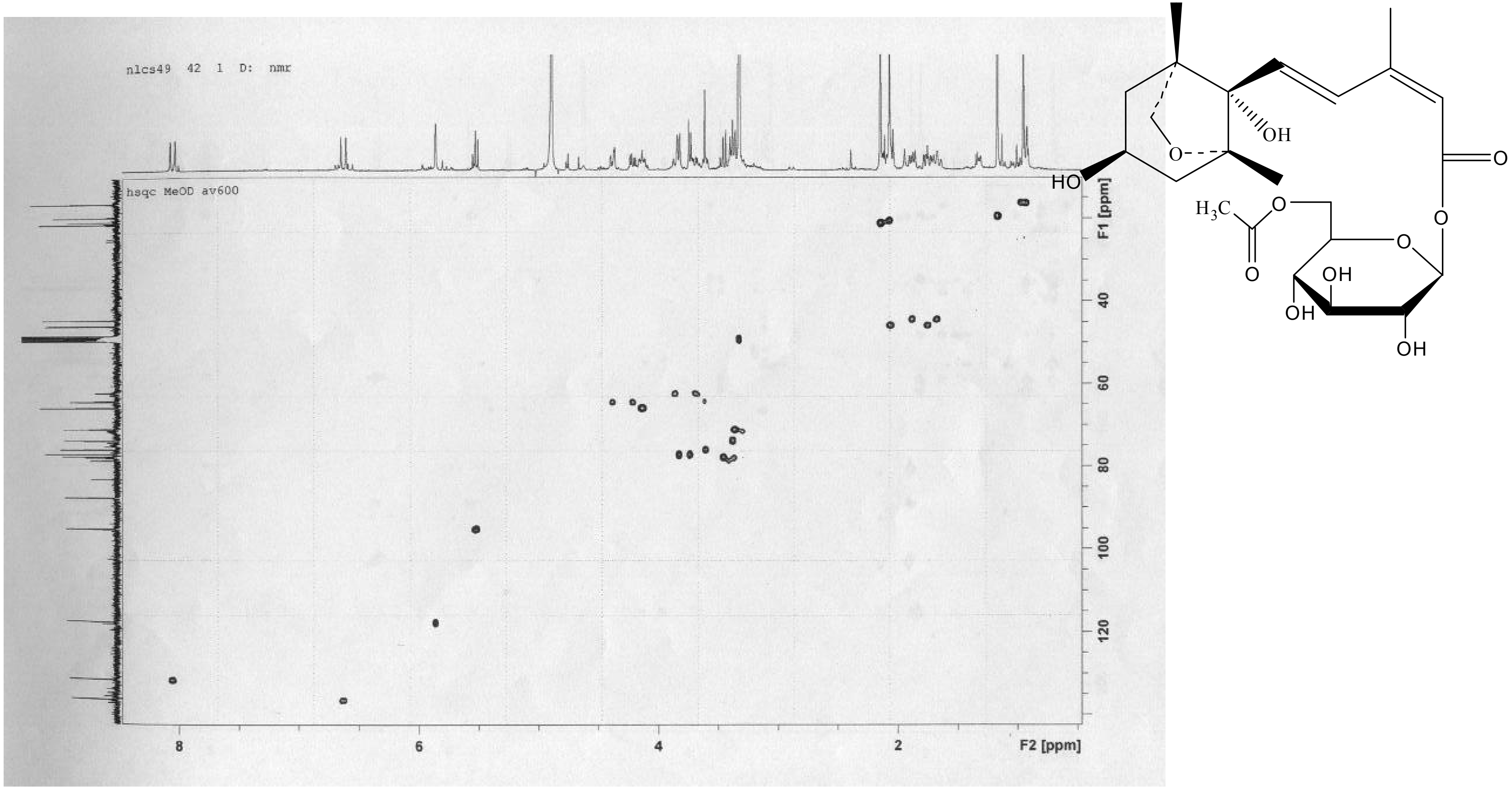

HMBC data of **2**

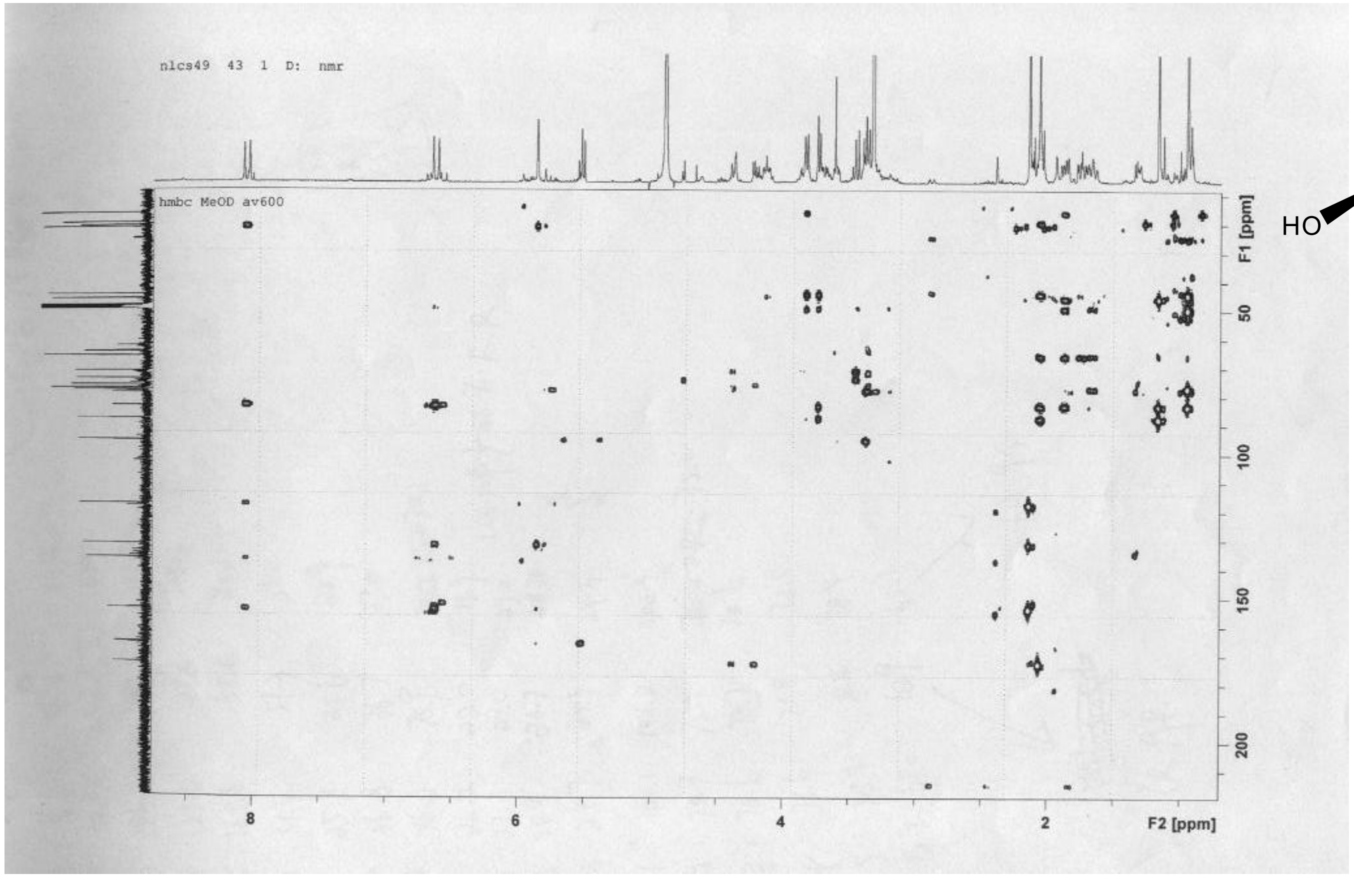

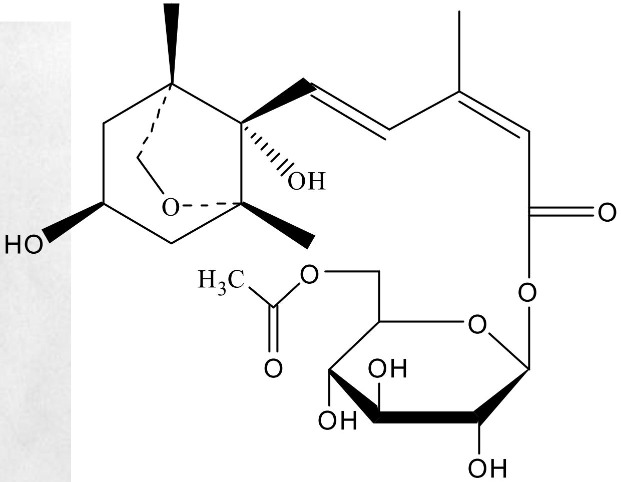

ROESY data of **2**

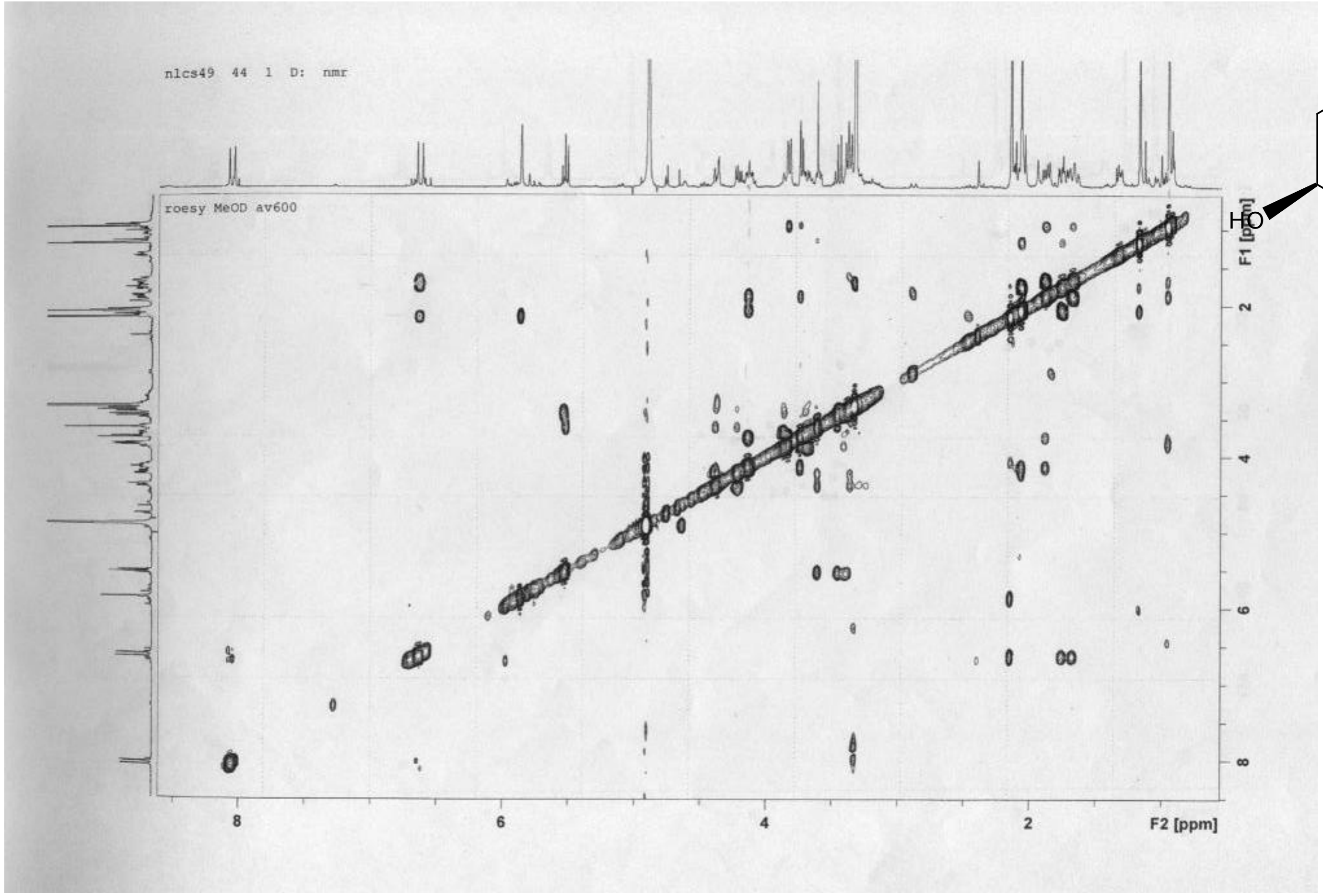

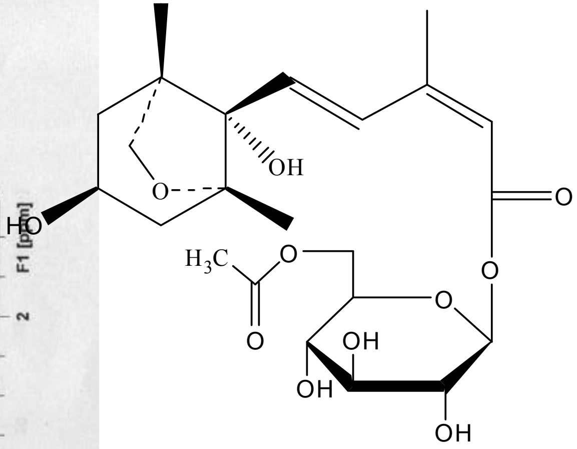

ESIMS data of **2**

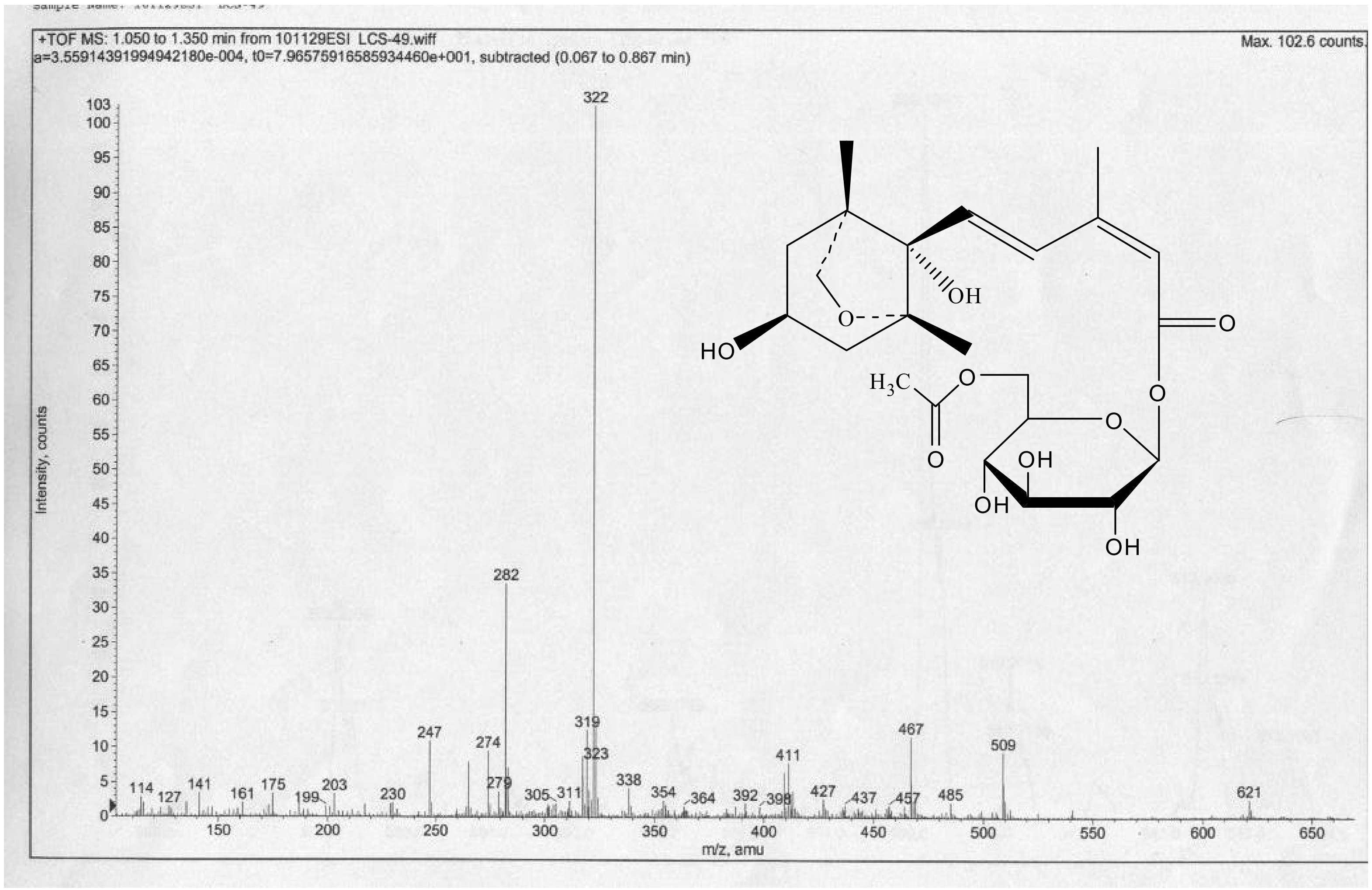

HRESIMS data of **2**

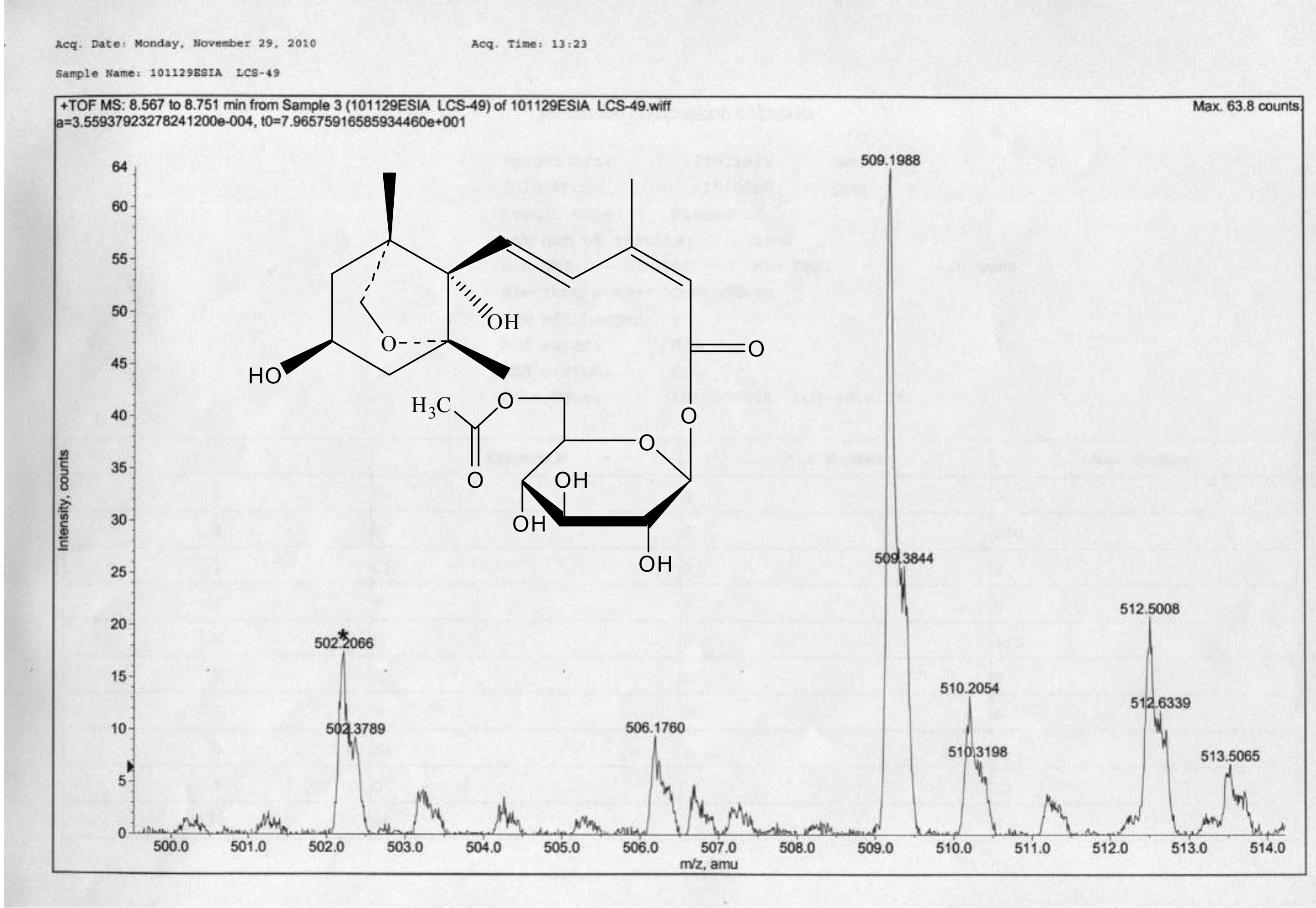

HRESIMS data of **2**

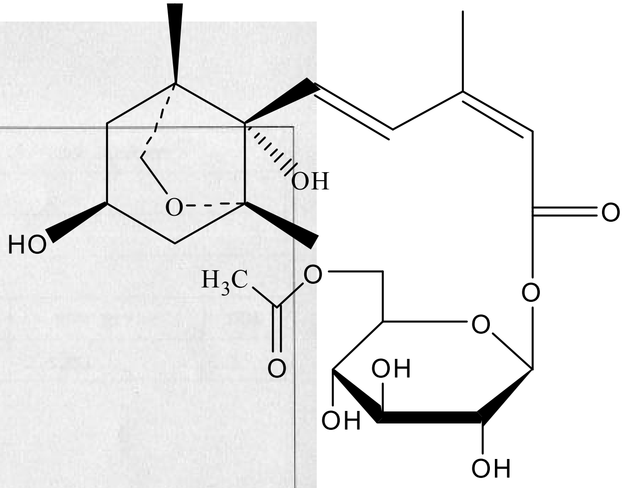

Acq. Date: Monday, November 29, 2010   Acq. Time: 13:23

Sample Name: 101129ESIA LCS-49

Elemental composition calculator

Target m/z: +509.1988 amu
Tolerance: +10.0000 ppm
Result type: Elemental
Max num of results: 1000
Min DBE: -10.0000 Max DBE: +60.0000
Electron state: OddAndEven
Num of charges: 0
Add water: N/A
Add proton: N/A
File Name: 101129ESIA LCS-49.wiff

	Elements	Min Number	Max Number
1	Br	0	0
2	C	0	200
3	Cl	0	0
4	F	0	0
5	H	0	400
6	K	0	0
7	N	0	0
8	Na	1	1
9	O	10	12
10	Pt	0	0

HRESIMS data of **2**

Acq. Date: Monday, November 29, 2010 Acq. Time: 13:23

Sample Name: 101129ESIA LCS-49

	Elements	Min Number	Max Number
11	S	0	0
12	Si	0	0

	Formula	Calculated m/z (amu)	mDa Error	PPM Error	DBE
1	C23 H34 O11 Na	509.1998	-1.0820	-2.1251	6.5

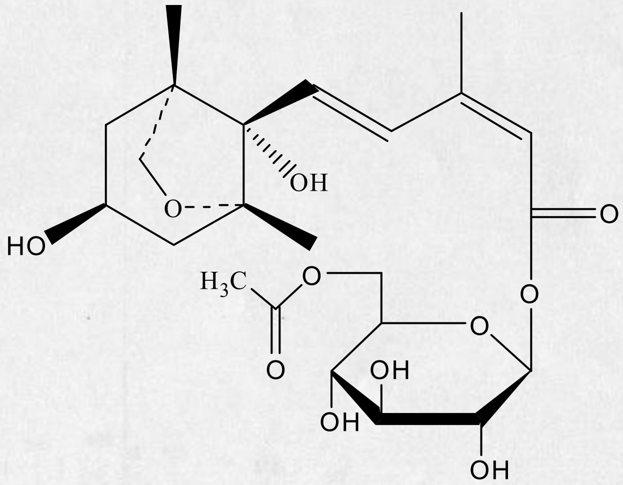

Supplement: Supplementary file 1 — Supplementary material, approximately 7.00 MB. [file 13659_2012_9_MOESM1_ESM.pdf]
